# DNA damage induced by KP372-1 hyperactivates PARP1 and enhances lethality of pancreatic cancer cells with PARP inhibition

**DOI:** 10.1038/s41598-020-76850-4

**Published:** 2020-11-19

**Authors:** Talysa Viera, Praveen L. Patidar

**Affiliations:** Department of Chemistry, New Mexico Institute of Mining and Technology, 801 Leroy Pl, Socorro, NM 87801 USA

**Keywords:** Biochemistry, Cancer, Cell biology, Drug discovery, Molecular biology

## Abstract

The overall prognosis for pancreatic cancer remains dismal and potent chemotherapeutic agents that selectively target this cancer are critically needed. Elevated expression of NAD(P)H:quinone oxidoreductase 1 (NQO1) is frequent in pancreatic cancer, and it offers promising tumor-selective targeting. Recently, KP372-1 was identified as a novel NQO1 redox cycling agent that induces cytotoxicity in cancer cells by creating redox imbalance; however, the mechanistic basis of KP372-1-induced cytotoxicity remains elusive. Here, we show that KP372-1 sensitizes NQO1-expressing pancreatic cancer cells and spares immortalized normal pancreatic duct cells, hTERT-HPNE. Notably, we found that KP372-1 is ~ 10- to 20-fold more potent than β-lapachone, another NQO1 substrate, against pancreatic cancer cells. Mechanistically, our data strongly suggest that reactive oxygen species produced by NQO1-dependent redox cycling of KP372-1 cause robust DNA damage, including DNA breaks. Furthermore, we found that KP372-1-induced DNA damage hyperactivates the central DNA damage sensor protein poly(ADP-ribose) polymerase 1 (PARP1) and activates caspase-3 to initiate cell death. Our data also show that the combination of KP372-1 with PARP inhibition creates enhanced cytotoxicity in pancreatic cancer cells. Collectively, our study provides mechanistic insights into the cytotoxicity instigated by KP372-1 and lays an essential foundation to establish it as a promising chemotherapeutic agent against cancer.

## Introduction

Pancreatic cancer has one of the highest mortality rates of all major cancers. It is predicted to be the second deadliest cancer within the next decade in the Western world^1,2^. It is often diagnosed in advanced stages where surgical removal is no longer an option for the majority of patients. Almost 80% of patients die within the first year of diagnosis in the United States. Patients undergoing radiation therapy and/or chemotherapy eventually develop refractory cancers. Currently for all diagnostic stages, the overall 5-year survival of these patients is less than 10%, which has remained largely unchanged in last 4 decades. Conventional therapies are subject to innate resistance and do not utilize pancreatic cancer-specific targets. Thus, chemotherapeutic agents selectively targeting pancreatic cancer are critically needed.

Elevated expression of NAD(P)H:Quinone Oxidoreductase 1 (NQO1) is frequent in pancreatic cancer, allowing for tumor-selective targeting, and NQO1-bioactivatable compounds have been shown to be highly effective against lung and pancreatic cancer^3–6^. NQO1 is a flavoprotein that functions as a homodimer, and each monomer that is bound to FAD catalyzes an obligatory two-electron reduction of a wide variety of quinones to their hydroquinone forms at the expense of cellular cofactors NADH or NADPH^7,8^. These hydroquinone forms are generally very unstable, spontaneously react with oxygen, and are converted back to parent quinones. This futile cycle causes significant NAD(P)H oxidation and generates reactive oxygen species (ROS) including superoxides that eventually leads to the formation of hydrogen peroxide (H_2_O_2_)^3–8^. The production of H_2_O_2_ creates oxidative stress and promotes cell death. Importantly, the majority of solid cancers including lung, colon, breast, and pancreatic cancer express elevated levels of NQO1, and these same tumors have significantly lowered Catalase (an H_2_O_2_ detoxifying enzyme) levels^5,6^. Consequently, the capacity of NQO1 to produce cytotoxic hydroquinones and alter the cellular redox state exclusively in cancer cells has emerged as an effective strategy to target cancers. Targeting NQO1:Catalase ratios of pancreatic cancers requires selective and potent NQO1 bioactivatable compounds.

One of the most studied NQO1 bioactivatable drugs is β-lapachone (β-lap). NQO1-dependent redox cycling of β-lap creates ROS that induces DNA damage and consequently hyperactivates the central DNA damage sensor protein poly(ADP-ribose) polymerase 1 (PARP1)^3–6,9^. Elevated PARP1 activity considerably reduces cellular NAD+ and ATP levels that eventually impede repair of DNA lesions caused by β-lap exposure and ultimately promotes cell death^10–13^. Collectively, the mechanism of β-lap-induced cytotoxicity is well understood in a variety of cancers, and it showed good efficacy in a phase 1 clinical trial (ARQ761, NCT02514031). However, dose-limiting anemia and methemoglobinemia remain the major challenges with β-lap as a monotherapy or in combination with other agents^14–16^.

Recently, KP372-1 was identified as a novel NQO1-bioactivatable compound that generates reactive oxygen species (ROS) and exhibits ~ 10 times greater anti-tumor activity than β-lap^17^. Also, KP372-1 apparently did not show any toxicity at doses that are required to produce anti-tumor activity in mice^17^. However, cellular consequences, specifically the DNA damage and repair response triggered by this compound, remain unknown. Moreover, initially, KP372-1 was identified as an inhibitor of Akt kinase^18–21^; however, Zhao and co-workers challenged this idea and proposed that KP372-1 contains quinone mimic moieties, and the NQO1-catalyzed reaction can reduce it to its ‘‘hydro’’ form using NAD(P)H via a two-electron reduction^17^. Thus, prior to exploring the clinical utility of KP372-1, it is necessary to define the underlying mechanistic basis of cellular consequences, specifically its potential to induce DNA damage response.

We hypothesize that KP372-1 is a better drug candidate than β-lap because NQO1-dependent redox cycling of KP372-1, as a single agent or combined with clinically relevant PARP inhibitors (PARPi), creates deleterious DNA lesions at a much lower dose, initiates DNA damage response, promotes cell death and offers a promising strategy to target pancreatic cancer. Here, we investigated this hypothesis by determining the suitability of targeting NQO1-expressing pancreatic cancer cells via KP372-1 and illustrated a previously unidentified mechanistic basis of cytotoxic effects of KP372-1 through instigating DNA damage response. We also explored the effectiveness of KP372-1 + PARPi combination to enhance the cytotoxicity of pancreatic cancer cells.

## Materials and methods

### Chemicals

KP372-1, β-lapachone (β-lap), dicoumarol (DIC), N-acetylcysteine amide (NAC), 3-(4,5-dimethylthiazol-2-yl)-2,5-diphenyltetrazolium bromide (MTT), Phenylarsine oxide (PAO) and Hoescht 33258 dye were purchased from Sigma-Aldrich (St. Louis, MO, USA). KP372-1 was also purchased from Echelon Biosciences Inc. (Salt Lake City, UT, USA) for comparison. KP372-1 from both sources worked in an identical manner for all the assays. BMN 673 (Talazoparib) was purchased from Selleck Chemicals LLC (Houston, TX, USA).

### Antibodies and siRNA

NQO1 (A180), PARP1 (F-2), total Akt (B1), anti-rabbit IgG-HRP (sc-2030) antibodies, and siNQO1 were purchased from Santa Cruz Biotechnology (Dallas, TX, USA). γH2AX (Clone JBW301) and anti-mouse IgG-HRP (AP160P) antibodies were obtained from EMD Millipore (Burlington, MA, USA). Cleaved caspase-3 (#9664S) and phosphoAkt (Ser473, D9E) antibodies were purchased from Cell Signaling Technology (Danvers, MA, USA). PAR (4335-MC) antibodies were obtained from Trevigen, (Gaithersburg, MD, USA). Non-target control siRNA (siSCR) and α-tubulin (T9026) antibodies were purchased from Sigma-Aldrich (St. Louis, MO).

### Oncomine data acquisition

Several pancreatic cancer mRNA expression profile data sets, indicated in the “Results” section, were downloaded from a public database, ONCOMINE (https://www.oncomine.org)^22^. Data sets were used directly since they have already been processed and normalized.

### Cell culture and siRNA transfection

All of the pancreatic cancer cell lines used in this study were a generous gift from the Der lab (University of North Carolina, Chapel Hill). MIA PaCa-2 and PANC-1 cells were maintained in DMEM (Lonza, Walkersville, MD, USA) supplemented with L-glutamine and 10% FBS. AsPC-1 and BxPC-3 cells were maintained in RPMI 1640 (Gibco, Life Technologies, Waltham, Massachusetts, USA) supplemented with 10% FBS. Capan-2 cells were maintained in McCoy’s 5A media (ATCC, Manassas, VA, USA) supplemented with 10% FBS. Finally, hTERT-HPNE cells were maintained in 75% DMEM and 25% Medium M3 Base (INCELL Corporation LLC, San Antonio, TX, USA) supplemented with 5% FBS, 10 ng/ml human recombinant EGF, 5.5 mM d-glucose (1 g/L), and 750 ng/ml puromycin. All cells were kept in a 37 °C incubator and 5% CO_2_. Cells were routinely monitored to confirm the absence of mycoplasma contamination.

For transient transfections, OptiMEM, Lipofectamine 2000 RNAiMax, siSCR, siNQO1 were used. Typical transfection experiments were done in 6-well plates (200,000 cells/well) using two sequential transfections to ensure higher efficiency of knockdown, each with 25 nM siRNAs for a total of 72 h. For experiments describing cell survival after NQO1 knockdown, plating and treatment with KP372-1 were completed within 72 h of the first transfection.

### Western blotting

For a typical Western blotting experiment, ~ 1 × 10^6^ cells were seeded in 35 mm dishes and allowed to adhere overnight. The next day, where appropriate, cells were treated with indicated concentrations (µM) of KP372-1 or KP3721 + dicoumarol (DIC) for the specified time points and H_2_O_2_ treatment (1 mM, 15 min in 1× PBS) was used as positive control. Cells were then lysed in ice-cold RIPA buffer (AlfaAesar, Haverhill, MA, USA) supplemented with 1× protease and 1× phosphatase inhibitors (Thermo Scientific, Ward Hill, MA, USA). Whole-cell extracts were sonicated, centrifuged at 13,000 rpm and supernatants were collected. Protein concentrations of supernatants were determined by the BCA assay (Thermo Scientific, Rockford, IL, USA). Proteins (15–20 µg) were separated by SDS-PAGE gels and transferred to nitrocellulose membranes. The blots were then blocked in either 1× casein blocking buffer (Sigma-Aldrich, St. Louis, MO, USA) or in 5% Skim Milk-TBST and incubated with primary antibodies followed by appropriate secondary antibody conjugated with HRP. Protein bands were detected by SuperSignal West Pico PLUS Chemiluminescent Substrate (Thermo Scientific, Rockford, IL, USA) and imaged on an Azure c600 (Azure Biosystems, Dublin, CA, USA). Blot images were adjusted for brightness and contrast, and were cropped to make final figures. For quantification of western blots, protein band intensities were analyzed using NIH ImageJ software (version 1.53c, https://imagej.net) and specific protein band intensities were normalized to the loading control. The reported relative intensities are the results of n ≥ 3. Raw data for all the Western blot images are provided in the supplementary information.

### Cell survival assays

#### DNA content assay

A modified cell survival assay measuring DNA content over ~ 7-day period was utilized^23^. Cells were seeded at 10,000 cells/well in 48-well plates in 0.5 ml of media. The next day, media were aspirated and replaced with 0.5 ml media containing the indicated concentrations of KP372-1 (µM) alone or in combination with 50 μM DIC. The cells were exposed for 2 hours (2 h) and the media were again aspirated and replaced with fresh media (without KP372-1). The cells were then allowed to grow for ~ 7 days or until control samples became confluent. Cells were then lysed in 250 µl dI water, freeze-thawed followed by suspension in 0.5 ml 1× TNE buffer containing Hoescht 33258 fluorescent dye and the DNA content was determined by measuring florescence signal using a Victor X5 plate reader (PerkinElmer, Waltham, MA, USA). Fluorescence values of treated samples were normalized to that of control samples and plotted as means ± SEM for treated over control (i.e., DMSO) treated (T/C) samples. The reported values are the results of the following sample sizes for KP372-1 ± DIC: MIA PaCa-2 concentration (n = 5); Capan-2 concentration (n = 3); MIA PaCa-2 ± siSCR/siNQO1 (n = 3); hTERT-HPNE (n = 3); PANC-1 (n = 3); AsPC-1 (n = 4); BxPC-3 (n = 3); MIA PaCa-2 time (n = 3); Capan-2 time (n = 3). The reported values are the results of the following sample sizes for β-lap ± DIC: MIA PaCa-2 (n = 4) and Capan-2 (n = 4).

#### Colony forming assay

MIA PaCa-2 or PANC-1 cells were seeded on 6-well plates at 250, 100, or 50 cells per well. The next day, cells were treated with vehicle (0.05% DMSO), 0.15 µM KP372-1, or 0.15 µM KP372-1 with 50 µM DIC for 2 h. The media was then replaced with fresh media and the cells were allowed to grow for 10 days. Next, the media was removed, and the colonies were fixed and stained with crystal violet solution containing 1× PBS, 1% formaldehyde, 1% methanol, and 0.05% w/v crystal violet for 20 min (min). The dishes were thoroughly rinsed in water and allowed to air dry. Colonies containing > 50 normal looking cells were and data (means ± SD) were expressed as treated/control (T/C) from experiments performed at least three times in triplicate. *p* values were obtained using an ordinary one-way ANOVA with Dunnett’s multiple comparisons test. The reported values are the results of n = 4.

#### MTT assay

Standard MTT assay protocol was followed with the following specifications^24^. Briefly, MIA PaCa-2, Capan-2, or PANC-1 cells were seeded in 96-well plates (4000 cells/well) and adhered overnight. The next day, cells were treated with the indicated concentrations of KP372-1, 50 µM DIC, or KP372-1 + 50 µM DIC for 2 h, followed by replacement with fresh media, and the cells were allowed to recover for 48 h. Phenylarsine oxide (PAO) was used as a positive control at a final concentration of 100 µM and 0.2% DMSO was used as a negative control. Following the 48 h recovery, 20 µl of MTT solution (5 mg/ml in 1× PBS) was added to each well and cells were incubated at 37 ºC for 2 h. Next, the supernatants were aspirated and 100 µL of DMSO was added to each well to dissolve the formazan crystals. Absorbance was then measured using a Victor X5 plate reader (PerkinElmer, Waltham, MA, USA). Data (%means ± S.D.) were expressed as treated/control values from three biological replicates. The reported values are the results of n = 4. *p* values were obtained using an ordinary one-way ANOVA with Dunnett’s multiple comparisons test.

### Reactive oxygen species (ROS) measurement

For the detection of H_2_O_2_ production, the ROS-Glo H_2_O_2_ assay kit (Promega, Madison, WI, USA) was used according to manufacturer’s recommendation with the indicated changes. Briefly, 15,000 cells/well were seeded in 96-well white-walled plates with clear bottoms and cells were allowed to adhere overnight. The following day, cells were treated with indicated concentrations (µM) of KP372-1 or KP372-1 + DIC or KP372-1 + N-acetylcysteine amide [NAC, 1 mM or 5 mM for total of 5 h (pre-treatment for 3 h and co-treatment for 2 h)] or DMSO (as control) for specified time (min) points in a total volume of 50 µl that contained 10 µl of H_2_O_2_ substrate. Then, 50 µl of ROS-Glo detection solution was added to each well and cells were incubated for 20 min at room temperature. Luminescence was measured using a Victor X5 plate reader. Luminescence values of treated samples were normalized to luminescence values of control samples to generate reported graphs. The reported values are the results of n = 4.

### 8-Oxoguanine (8-oxoG) measurement

Cells were seeded on 6-well plates (~ 200,000 cells/well) containing glass slides and allowed to adhere overnight. The next day, cells were treated with indicated concentrations (µM) of KP372-1 or KP372-1 + DIC or KP372-1 + N-acetylcysteine amide [NAC, 5 mM for total of 4 h (pre-treatment for 3 h and co-treatment for 1 h)] or DMSO (as control) for 1 h. Cells treated with H_2_O_2_ (1 mM, 15 min in 1× PBS) served as positive control. Afterwards, media were replaced with fresh media (without KP372-1). Next, standard immunofluorescence microscopy protocol was followed as described previously^25^. Briefly, cells were gently washed in 1× PBS, followed by fixation with ice-cold methanol:acetic acid (3:1, v/v) overnight at − 20 °C. Fixed cells were gently washed in 1× PBS at room temperature (3×, 5 min each) followed by incubation in blocking solution (1× PBS containing 5% normal goat serum) for 1 h at room temperature. Next, the cells were incubated with 8-oxoG primary antibody (1:2000 dilution in 1× PBS containing 5% normal goat serum) for 1 h at room temperature. The cells were then washed (3×, in 1× TBST followed by 1× in PBST, 5 min each) and incubated with Alexa Fluor 594 fluorescent secondary antibody (1:2000 dilution in 1× PBS containing 5% normal goat serum) for 1 h at room temperature. The cells were then washed (3×, in 1× TBST followed by 1× in PBST, 5 min each). Finally, the wash buffer was removed, and the cover glass was mounted with prolong gold antifade mounting medium with DAPI (nuclear stain). Images were acquired using Olympus FV10i confocal laser scanning microscope with 60× oil immersion objective. The images were analyzed and quantified using NIH ImageJ software (version 1.53c, https://imagej.net). Reported data is representative of n = 4, each in duplicate from total of 150 cells. *p* values were obtained using an ordinary one-way ANOVA with Dunnett’s multiple comparisons test.

### Neutral comet assay

For the neutral comet assay, the Comet Assay Kit (Trevigen, Gaithersburg, MD, USA) was used according to manufacturer’s recommendation with the indicated changes. Briefly, MIA PaCa-2 cells were plated on 6-well plates and allowed to adhere overnight. The next day, cells were treated for 1 h with vehicle control (0.05% DMSO), 0.15 µM KP372-1, 0.15 µM KP372-1 with 50 µM DIC, or 0.15 µM KP372-1 with N-acetylcysteine amide [NAC, 5 mM for total of 4 h (pre-treatment for 3 h and co-treatment for 1 h)]. 1 mM H_2_O_2_ in PBS treated for 15 min was used as a positive control. Cells were trypsinized and collected, washed with PBS, and resuspended in PBS at a concentration of 2 × 10^5^ cells/mL. Cells were added to melted LMAgarose (Trevigen) cooled to 37 °C at a ratio of 1:10 and pipetted onto a pre-warmed comet slide and spread evenly. Slides were then placed at 4 °C for 30 min to allow adherence of the agarose to the slides. The slides were then gently immersed in lysis solution overnight at 4 °C. Following lysis, the slides were immersed in 1× Neutral Electrophoresis Buffer containing tris base and sodium acetate (corrected to pH 9 with glacial acetic acid) for 30 min at 4 °C. The slides were then electrophoresed at 20 V for 45 min at 4 °C in the neutral electrophoresis buffer. Next, the slides were gently immersed in DNA precipitation solution containing 1 M ammonium acetate in 95% ethanol for 30 min at room temperature followed by immersion in 70% ethanol for 30 min at room temperature. The slides were then dried at 37 °C for 10 min and subsequently stained with a 1:25,000 dilution of SYBR Green in TE buffer (10 mM Tris–HCl pH 7.5 with 1 mM EDTA) for 30 min at room temperature in the dark. Slides were rinsed briefly with distilled water twice and then allowed to fully dry before imaging. Images were acquired using an Olympus FV10i confocal laser scanning microscope with a 10× objective. The comets were analyzed using the ImageJ (version 1.53c, https://imagej.net) plug-in OpenComet v1.3 (www.biocomet.org) and the tail moment was normalized to the DMSO control (n = 100 comets per sample). *p* values were obtained using an ordinary one-way ANOVA with Dunnett’s multiple comparisons test.

#### Confocal immunofluorescence microscopy

The general procedure for confocal immunofluorescence microscopy is similar as described previously^25^. Briefly, cells were seeded on 6-well plates (~ 100,000 cells/well) containing glass slides and allowed to adhere overnight. The next day, cells were treated with DMSO or KP372-1 (0.15 µM) for 2 h. Afterwards, media were replaced with fresh media (without KP372-1). Then, cells were fixed at indicated time points (24, 48, 72 and 96 h) by gentle washing in 1× PBS, followed by fixation with ice-cold methanol:acetic acid (3:1, v/v) overnight at − 20 °C. Fixed cells were gently washed in 1× PBS at room temperature (3×, 5 min each). The cells were then incubated in blocking solution (1× PBS containing 5% normal goat serum) for 1 h at room temperature. Then, the cells were incubated with cleaved caspase-3 (i.e., activated caspase) primary antibody (1:500 dilution in 1× PBS containing 5% normal goat serum) for overnight at 4 °C. The next day, the cells were washed (3×, 5 min each in 1× PBS) and incubated with Alexa Fluor 594 fluorescent secondary antibody (1:1000 dilution in 1× PBS containing 5% normal goat serum) for 1 h at room temperature. The cells were then washed (3×, 5 min each in 1× PBS). Finally, the wash buffer was removed, and the cover glass was mounted with prolong gold antifade mounting medium with DAPI (nuclear stain). Images were acquired using Olympus FV10i confocal laser scanning microscope with a 60× oil immersion objective.

#### Synergy calculations

Drug synergy was calculated using CompuSyn 1.0 software (www.combosyn.com). MIA PaCa-2 cells (4000 cells/well) were seeded in 96-well plates and treated with KP372-1 (2 h) or BMN 673 (24 h) alone to determine the IC_50_ values of each drug alone. Cells were treated with a non-constant combination of KP372-1 and BMN 673 for 2 h and replaced with media containing the same concentrations of BMN 673 for additional 22 h (24 h total). Then, cell survival was assessed via DNA content assay similarly to as described above. Relative survival was plotted to obtain Fraction affected (Fa) values. Fa values were normalized to the DMSO control and entered into CompuSyn. Dose-Reduction Index (DRI) values were calculated using the Chou-Talalay method^26–28^. Briefly, DRI values are calculated using the following equation

$$DRI = \frac{{(Dx)_{1} }}{{\left( D \right)_{1} }}$$

where (D_x_)_1_ is the dose of the drug alone and (D)_1_ is the dose of the drug in combination^26^. DRI values are defined as favorable (DRI > 1) or unfavorable (DRI < 1). Reported values are the results of n = 3.

#### Statistical analysis

Unless otherwise stated, data (mean ± SEM) were graphed and two-tailed Student’s t tests using the Holm-Sidak method to correct for multiple (more than one) comparisons were performed. For the 8-oxoG experiments, the neutral comet assay, and colony forming assays, an ordinary one-way ANOVA was used to compare treated samples to control. The minimum biological replicate size was n = 3. Alpha was set to 0.05. GraphPad Prism 8 was used to perform statistical analyses. Images are representative results of experiments performed with n ≥ 3 biological repeats. *p < 0.05; **p < 0.01, ***p < 0.001, ****p < 0.0001.

## Results

### NQO1 expression is elevated in pancreatic cancer

**Figure 1:**
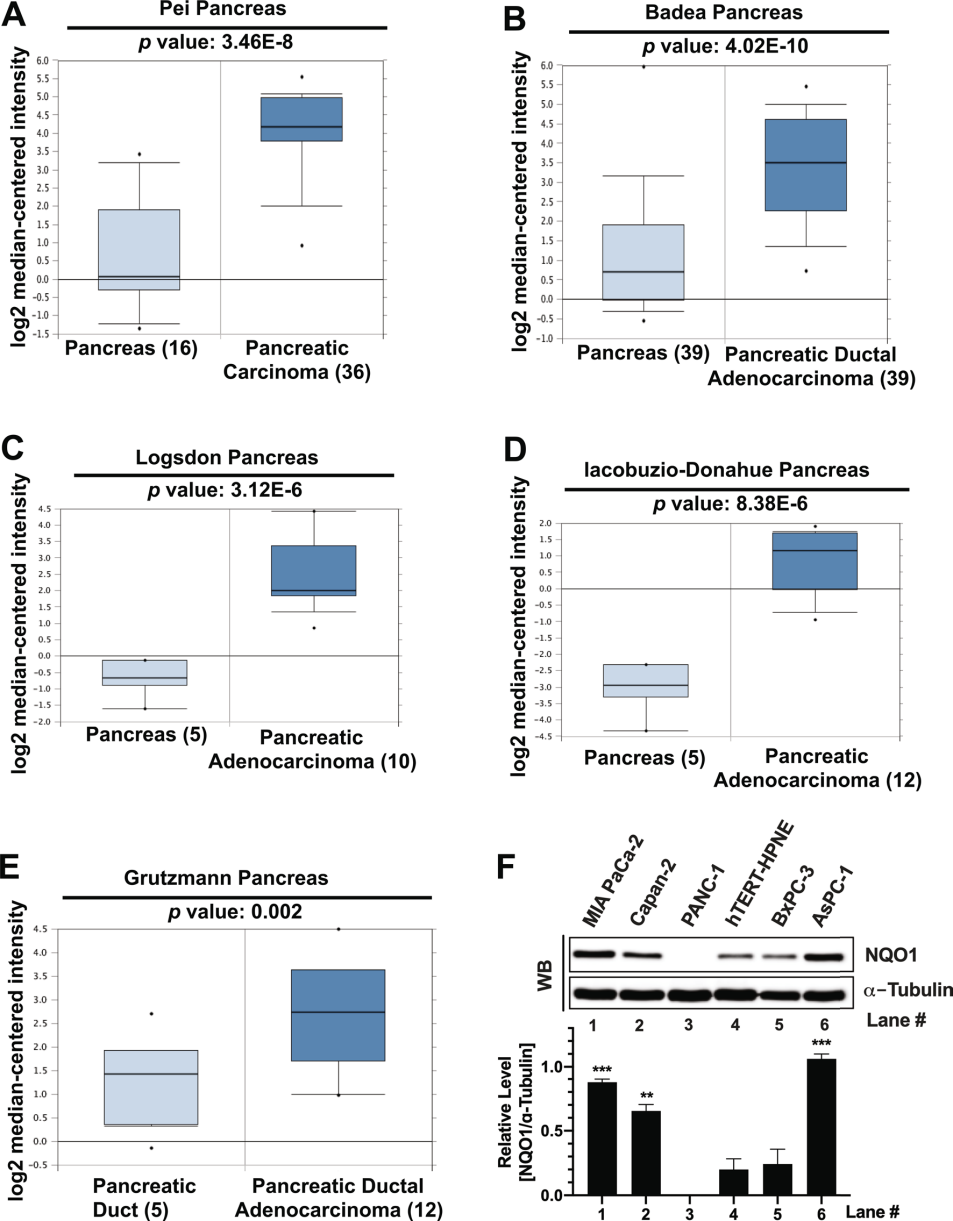
Upregulation of NQO1 in pancreatic cancer. (**A**–**E**) Data retrieved from ONCOMINE displaying changes in NQO1 mRNA levels (log2 median-centered) for pancreatic normal tissue compared to tumor tissue from indicated samples (in parentheses) with *p* values. (**F**) Top panel shows a representative image of Western blot analyses for NQO1 protein level in frequently utilized pancreatic cancer cell lines along with non-cancerous pancreatic duct cells (hTERT-HPNE). α-Tubulin was used as a protein loading control. Bottom panel shows quantification of band intensity detected by ImageJ software (version 1.53c, https://imagej.net) for NQO1 normalized to α-tubulin of each respective sample. Graphs represent means ± SEM from n = 4. *p* values were obtained via two-tailed student’s t-tests. *p < 0.05; **p < 0.01; ***p < 0.001, comparing pancreatic cancer cell lines with hTERT-HPNE. WB; Western blot. Raw data for all the Western blot images are provided in the supplementary information.

NQO1 overexpression has been reported in a variety of solid tumors including lung and pancreatic cancer, and targeting NQO1 has emerged as a promising strategy^3–6,29^. To further determine the suitability of NQO1 as a potential target against pancreatic cancer, we utilized the Oncomine database to evaluate its expression. Within the database, we found that multiple studies reported significantly elevated NQO1 mRNA levels, ranging from three to tenfold, in pancreatic cancer compared to normal pancreatic tissue from a considerable number of patients (Fig. 1A–E). Overall, 109 pancreatic cancer specimens show significantly higher level of NQO1 expression compared to 70 normal pancreatic tissue (a total of 179 specimens).

To strengthen the suitability of NQO1 as a promising target against pancreatic cancer, we evaluated NQO1 protein levels in five different commonly utilized model pancreatic cancer cell lines and one non-cancerous, immortalized pancreatic duct cell line, hTERT-HPNE. We found that MIA PaCa-2, Capan-2 and AsPC-1 show significantly higher NQO1 protein levels compared to hTERT-HPNE (Fig. 1F). Whereas, BxPC-3 showed a similar NQO1 level to that of hTERT-HPNE, and PANC-1 did not show any detectable NQO1 expression (Fig. 1F). The absence of detectable level of NQO1 protein in PANC-1 cells is consistent with a previous study reporting a polymorphism in NQO1 gene leading to enhanced susceptibility of expressed NQO1 to proteasome-mediated degradation in these cells^30,31^. Collectively, data presented in Fig. 1 strongly suggest that elevated NQO1 levels offer a promising target for therapeutic intervention against pancreatic cancer.

### Elevated NQO1 expression sensitizes pancreatic cancer cells to KP372-1

**Figure 2:**
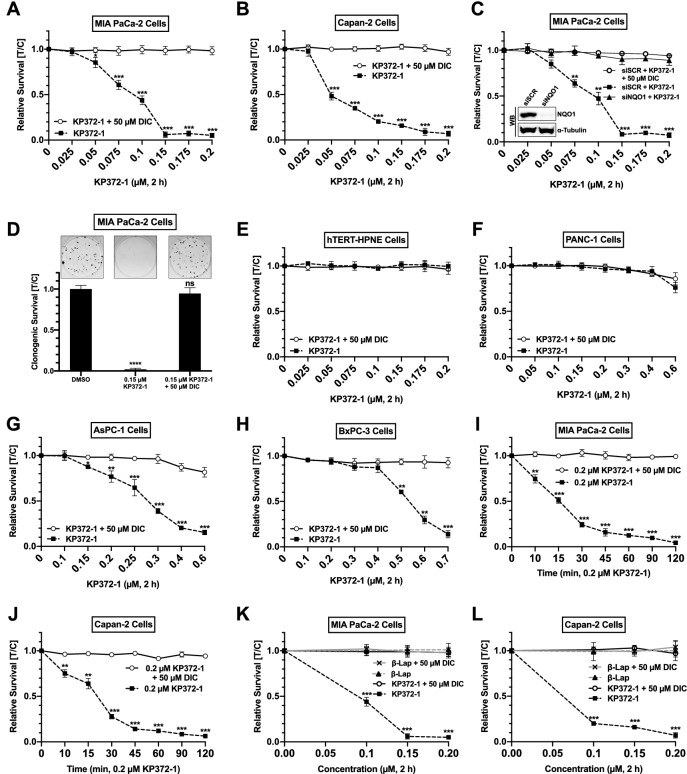
Elevated NQO1 expression sensitizes pancreatic cancer cells to KP372-1. (**A**,**B**) Relative survival measured by DNA content assay in the presence of indicated concentrations (µM) of KP372-1 ± dicoumarol (DIC, NQO1 inhibitor) for 2 h. (**C**) Relative survival of siSCR or siNQO1 knockdown cells in the presence of indicated concentrations (µM) of KP372-1 ± DIC for 2 h. (**D**) Clonogenic survival in the presence of indicated concentrations (µM) of KP372-1 ± DIC for 2 h. (**E**–**H**) Relative survival in the presence of indicated concentrations (µM) of KP372-1 ± DIC for 2 h. (**I**,**J**) Relative survival in the presence of 0.2 µM concentration of KP372-1 ± 50 µM DIC for indicated time points. (**K**,**L**) Relative survival in the presence of KP372-1 ± 50 µM DIC and β-lap ± 50 µM DIC. Graphs represent means ± SEM for drug treatment over control (i.e., DMSO) treated (T/C) samples for MIA PaCa-2 n = 5 (**A**), Capan-2 n = 3 (**B**), MIA PaCa-2 ± siSCR/siNQO1 n = 3 (**C**), MIA PaCa-2: clonogenic survival n = 4 (**D**), hTERT-HPNE n = 3 (**E**), PANC-1 n = 3 (**F**), AsPC-1 n = 4 (**G**), and BxPC-3 n = 3 (**H**), MIA PaCa-2: time course (**I**), Capan-2: time course n = 3 (**J**), MIA PaCa-2: β-lap treatment from n = 4 (**K**), and Capan-2: β-lap treatment n = 4 (**L**). Each biological replicate was performed in triplicate. *p* values were obtained via two-tailed student’s t-tests. *p < 0.05; **p < 0.01; ***p < 0.001, comparing KP372-1 alone with KP372-1 + DIC or KP372-1 + β-lap. For the clonogenic survival assay, *p* values were obtained via an ordinary one-way ANOVA using the Dunnett’s multiple comparisons test. ****p < 0.0001; ns, not significant, comparing indicated drug treatments to the DMSO control.

In a previous study, KP372-1 was identified as a potent NQO1-mediated redox cycling agent^17^. Thus, we sought to systematically evaluate the cytotoxicity induced by KP372-1 against a panel of pancreatic cancer cell lines mentioned in Fig. 1 via the DNA content assay. We found that MIA PaCa-2 and Capan-2, two frequently used pancreatic cancer model cell lines, show robust toxicity with 2 h exposure to KP372-1 where concentrations as low as 0.05 µM caused significant cell death and a concentration of 0.2 µM led to > 95% cell death (Fig. 2A,B, respectively). Dicoumarol (DIC), an inhibitor of NQO1, rescued these cells completely from cytotoxic effects of KP372-1 (Fig. 2A,B). Importantly, the siRNA-mediated transient knockdown of NQO1 reversed the sensitivity of MIA PaCa-2 cells against KP372-1 and provided genetic evidence that KP372-1-induced cytotoxicity is NQO1-dependent (Fig. 2C). Similar to the DNA content assay, the clonogenic survival and MTT assays provided additional validation of robust toxicity induced by KP372-1 against pancreatic cancer cells that can be rescued by DIC (Figs. 2D and S1A,B, respectively). Notably, similar KP372-1 treatment conditions did not elicit cell death in the immortalized normal pancreatic duct cell line, hTERT-HPNE (Fig. 2E). Likewise, PANC-1 cells with no detectable level of NQO1 protein also did not show appreciable toxicity (Figs. 2F, S1C, and S2A). Furthermore, AsPC-1 and BxPC-3 cells showed significant toxicity at ≥ 0.15 µM and at ≥ 0.5 µM KP372-1 (Fig. 2G,H, respectively). Despite having high levels of NQO1, the sensitivity of AsPC-1 cells is lower compared to MIA PaCa-2 (Figs. 1F and 2A). This is likely due to ~ 2.5 times higher antioxidant capacity of AsPC-1 cells compared to MIA PaCa-2 cells^32^. Finally, to determine the minimum time of exposure required to induce cell death, we carried out a time-course experiments for MIA PaCa-2 and Capan-2 with 0.2 µM KP372-1. We found that merely 10 min exposure is sufficient to induce significant cell death, whereas 2 h exposure led to > 95% cell death in MIA PaCa-2 and Capan-2 (Fig. 2I,J, respectively). Next, we compared toxicity of KP372-1 with β-lapachone (β-lap) against some of the pancreatic cancer cells. For MIA PaCa-2 cells, β-lap did not induce appreciable toxicity up to 2.0 µM, whereas, 0.2 µM KP372-1 was lethal for these cells (Figs. 2K and S1D). For Capan-2 cells, β-lap did not induce appreciable toxicity up to 1.0 µM, whereas, 0.2 µM KP372-1 was lethal for these cells (Figs. 2L and S1E). These data clearly show that KP372-1 is much more potent (at least ~ 10- to 20-fold) than β-lap against pancreatic cancer cells. Overall, our data strongly suggest that the potent redox cycling agent KP372-1 selectively induces cell death in NQO1-expressing pancreatic cancer cells and spares non-cancerous immortalized pancreatic duct cells.

### KP372-1 treatment enhances ROS production in pancreatic cancer cells

**Figure 3:**
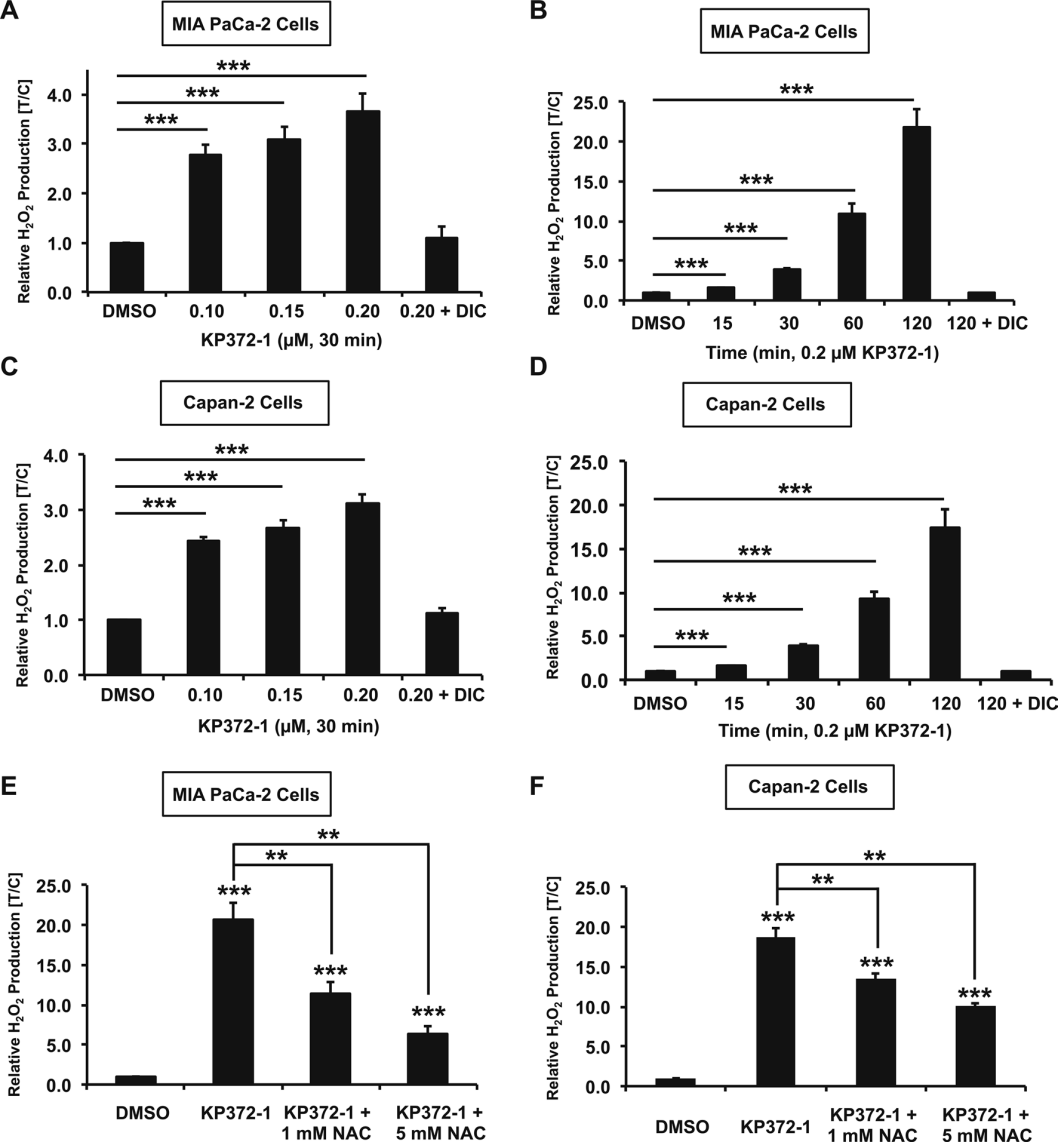
KP372-1 treatment enhances ROS production in pancreatic cancer cells. (**A**–**D**) Relative levels of H_2_O_2_ production in control (DMSO), KP 372-1 or KP372-1 ± 50 µM DIC treated cells were measured using the Promega ROS-Glo H_2_O_2_ assay kit. MIA PaCa-2 cells treated, with indicated dose of KP372-1 (µM) for 30 min (**A**), and with 0.2 µM KP372-1 for indicated time (min) points (**B**). Capan-2 cells treated, with indicated dose of KP372-1 (µM) for 30 min (**C**), and with 0.2 µM KP372-1 for indicated time (min) points (**D**). (**E**,**F**) Relative levels of H_2_O_2_ production in control (DMSO), KP372-1 and KP372-1 ± N-acetylcysteine amide [NAC, 1 mM or 5 mM for total of 5 h (pre-treatment for 3 h and co-treatment for 2 h)] treated MIA PaCa-2 cells (**E**) or Capan-2 cells (**F**) with indicated concentrations**.** Bar graphs represent means ± SEM for treated/control samples from n = 4, each performed in duplicate. *p* values were obtained via two-tailed student’s t-tests. *p < 0.05; **p < 0.01; ***p < 0.001, comparing treated with control samples.

To systematically define the mechanistic basis of cytotoxicity, we sought to evaluate the production of ROS due to NQO1 redox cycling of KP372-1 in pancreatic cancer cells. Specifically, we measured H_2_O_2_ formation in MIA PaCa-2 and Capan-2 cells and carried out dose–response and time-course studies. Compared to control (DMSO treatment), 30 min exposure of indicated concentrations (µM) of KP372-1 caused significant enhancement of H_2_O_2_ production in MIA PaCa-2 cells, whereas co-treatment of KP372-1 + DIC rescued ROS formation to control levels (Fig. 3A). Next, we carried out a time course response with indicated KP372-1 concentrations (µM) and found that H_2_O_2_ production is dramatically enhanced by 2 h treatment in MIA PaCa-2 cells (Fig. 3B). To further validate KP372-1-induced H_2_O_2_ production, we carried dose and time-course studies using Capan-2 cells and found similar enhancement of H_2_O_2_ formation in these cells (Fig. 3C,D, respectively). Finally, we utilized a well-known ROS scavenger, N-acetylcysteine amide (NAC) to rescue KP372-1-induced ROS production in pancreatic cancer cells. We found that NAC significantly rescues ROS production in a dose-dependent manner in both MIA PaCa-2 and Capan-2 cells treated with KP372-1 (Fig. 3E,F, respectively). Notably, KP372-1 treatment did not elicit ROS production above background levels in NQO1-deficient PANC-1 cells (Fig. S2B). Together, these data strongly support high levels of ROS production in NQO1-expressing pancreatic cancer cells after KP372-1 treatment.

### KP372-1 elicits robust DNA damage in pancreatic cancer cells

**Figure 4:**
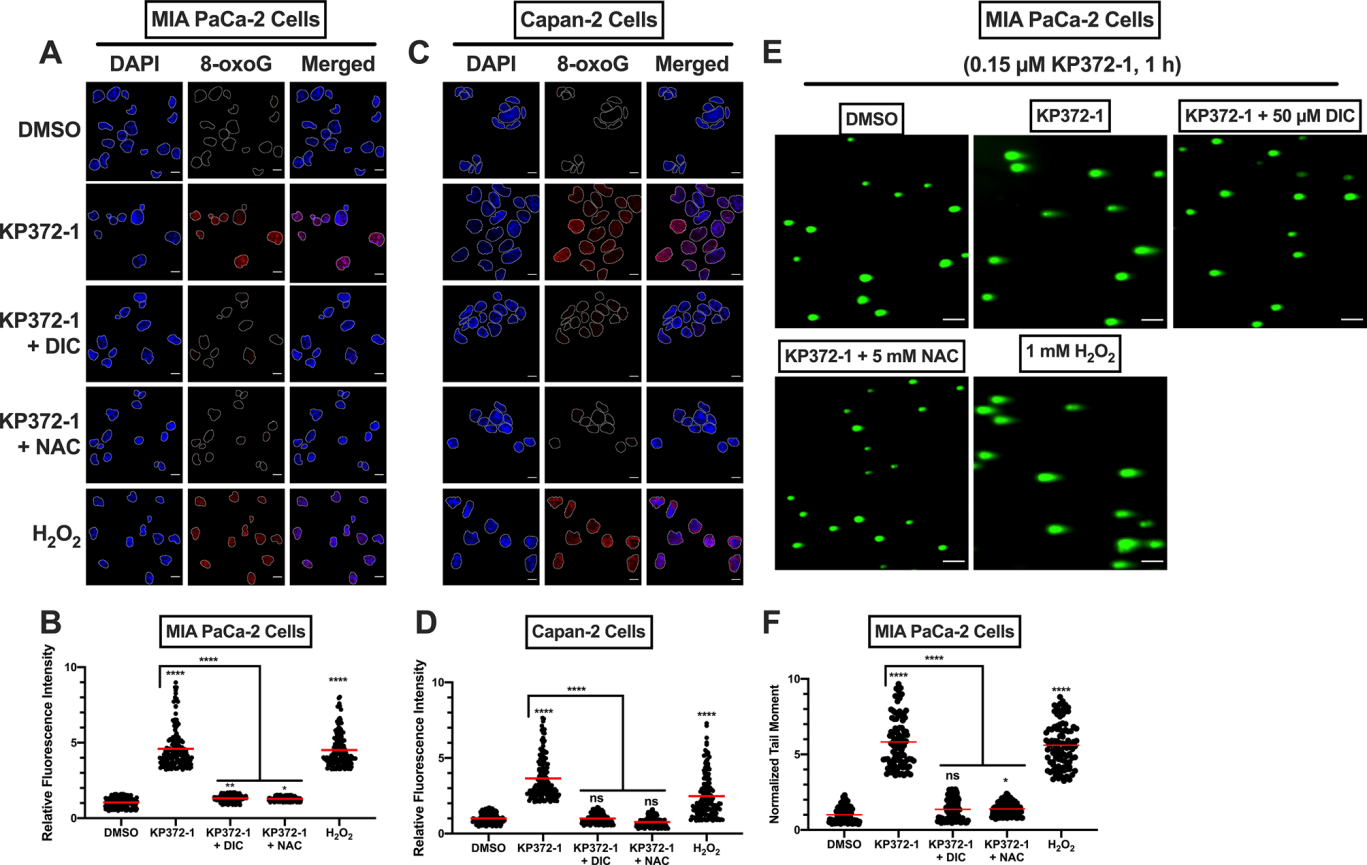
KP372-1 treatment instigates oxidative DNA damage and DNA breaks in pancreatic cancer cells. (**A**–**D**) Relative levels of nuclear 8-oxoG signal in control (DMSO), 0.15 µM KP372-1, 0.15 µM KP372-1 ± 50 µM DIC or KP372-1 ± N-acetylcysteine amide [NAC, 5 mM for total of 4 h (pre-treatment for 3 h and co-treatment for 1 h)] treated cells were measured by immunofluorescence confocal microscopy. Representative images of MIA PaCa-2 cells (**A**), and quantification of fluorescence signal (**B**). Representative images of Capan-2 cells (**C**), and quantification of fluorescence signal (**D**). Cells treated with H_2_O_2_ (1 mM, 15 min in 1× PBS) served as positive control. The scale bar represents 10 µm. Graphs represent the means (red bar) for treated/control samples from n = 3, each performed in duplicate for total of 150 cells. Relative fluorescence intensities were determined by ImageJ software (version 1.53c, https://imagej.net). (**E**,**F**) Relative levels of comet tail moment of control (DMSO), 0.15 µM KP372-1 ± 50 µM DIC or KP372-1 ± N-acetylcysteine amide [NAC, 5 mM for total of 4 h (pre-treatment for 3 h and co-treatment for 1 h)] treated cells measured by confocal microscopy. Representative images of MIA PaCa-2 cells (**E**), and quantification of fluorescence signal (**F**). Cells treated with H_2_O_2_ (1 mM, 15 min in 1× PBS) served as positive control. The scale bar represents 10 µm. Tail moments were obtained using the ImageJ (version 1.53c, https://imagej.net) plug-in OpenComet v1.3 (www.biocomet.org). Graphs represent the means (red bar) for treated/control samples from n = 3, each performed in duplicate for total of 150 cells. *p* values were obtained via an ordinary one-way ANOVA using the Dunnett’s multiple comparisons test. *p < 0.05; **p < 0.01; ****p < 0.0001; ns, not significant, comparing indicated drug treatments to the DMSO control.

The profound increase in ROS levels in pancreatic cancer cells strongly suggested that KP372-1 might cause DNA damage, including DNA breaks. To test this idea, we utilized a variety of approaches. First, we investigated direct oxidative DNA damage induced by KP372-1 treatment through measuring 8-oxoguanine (8-oxoG) levels detected by 8-oxoG specific antibodies via immunofluorescence confocal microscopy. MIA PaCa-2 cells showed robust 8-oxoG signal after 0.15 µM KP372-1 exposure for 1 h compared to control (DMSO-treated) cells, and treatment with DIC or the ROS scavenger NAC significantly reduced the 8-oxoG levels instigated by KP372-1, comparable to control levels (Fig. 4A,B). MIA PaCa-2 cells treated with H_2_O_2_ were used as positive control (Fig. 4A,B). To further support the 8-oxoG formation, we carried out similar studies in Capan-2 cells. As anticipated, we observed a strong induction of 8-oxoG signal by KP372-1 treatment that was rescued by DIC or NAC treatment (Fig. 4C,D). Importantly, KP372-1 treatment did not cause an increase in 8-oxoG signal above background levels in NQO1-deficient PANC-1 cells (Fig. S2C,D). These data strongly support oxidative DNA damage instigated by KP372-1 in NQO1-expressing pancreatic cancer cells.

Next, we examined the induction of DNA double strand breaks (DSBs) after KP372-1 treatment by utilizing the neutral comet assay. MIA PaCa-2 cells treated with 0.15 µM of KP372-1 for 1 h showed significantly elevated comet tail moments compared to control (DMSO-treated) cells (Fig. 4E,F). Importantly, treatment of DIC or NAC significantly reduced the comet tail moment of KP372-1-treated cells (Fig. 4E,F). MIA PaCa-2 cells treated with H_2_O_2_ served as positive control (Fig. 4E,F). These data provide clear evidence that KP372-1 treatment induces DSBs in NQO1-expressing pancreatic cancer cells.

**Figure 5:**
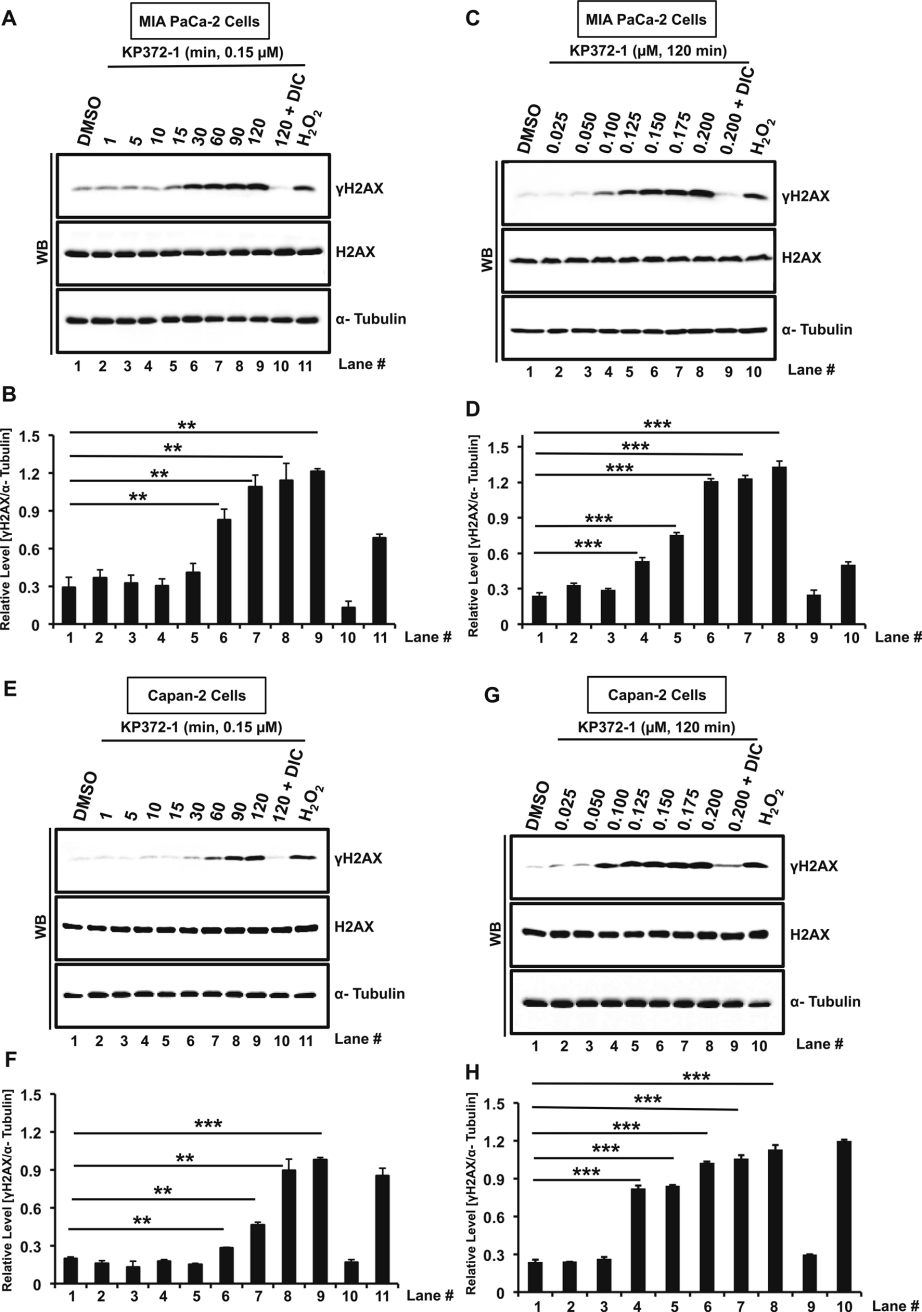
KP372-1 elicits robust DNA damage signaling in pancreatic cancer cells. (**A**–**H**) Assessment of phosphorylated H2AX (yH2AX) via Western blotting as a marker of DNA damage response induced by KP372-1. MIA PaCa-2 cells treated with 0.15 µM KP372-1 ± 50 µM DIC for indicated time (min) points (**A**,**B**), blot image and quantification, respectively, and with indicated dose of KP372-1 (µM) ± 50 µM DIC for 120 min (**C**,**D**), blot image and quantification, respectively. Capan-2 cells treated with 0.15 µM KP372-1 ± 50 µM DIC for indicated time (min) points (E–F), blot image and quantification, respectively, and with indicated dose of KP372-1 (µM) ± 50 µM DIC for 120 min (**G**,**H**), blot image and quantification, respectively. Representative Western blot images are presented. Bar graphs represent quantification of band intensities (means ± SEM) of yH2AX normalized to α-tubulin of respective sample from n = 5. Band intensities were detected by ImageJ software (version 1.53c, https://imagej.net) and α-tubulin was used as loading control. Cell lysates prepared from H_2_O_2_-treated cells (1 mM, 15 min in 1× PBS) served as positive control. *p* values were obtained via two-tailed student’s t-tests. *p < 0.05; **p < 0.01; ***p < 0.001, comparing treated with control (DMSO) samples. WB; Western blot. Raw data for all the Western blot images are provided in the supplementary information.

Finally, to provide additional validation, time-course and dose–response studies were carried out to evaluate alteration in phosphorylated H2AX (γH2AX) levels using Western blotting as a proxy of DSB formation and concomitant signaling after KP372-1 treatment. Cell lysate from H_2_O_2_ treated cells were utilized as positive control. A significant level of DNA damage was observed at multiple time points within a 2 h window in MIA PaCa-2 cells treated with 0.15 µM KP372-1 compared to control (DMSO treated) samples, where co-treatment with DIC eliminated DNA damage (Fig. 5A,B). Next, we carried dose–response experiments with 2 h of KP372-1 treatment and found a clear dose-dependent enhancement of DNA damage (Fig. 5C,D). To further validate DNA damage induction by KP372-1 treatment, similar studies were carried out in Capan-2 cells. As anticipated, we found clear time- and dose-dependent induction of DNA damage in Capan-2 cells (Fig. 5E–H, respectively) similar to that of MIA PaCa-2 cells. Similar treatment of NQO1-deficient PANC-1 cells did not show increased γH2AX signal above background levels (Fig. S2E). Collectively, these data strongly support a robust DNA damage response instigated by KP372-1 in NQO1-expressing pancreatic cancer cells.

### KP372-1 hyperactivates PARP1 in pancreatic cancer cells

**Figure 6:**
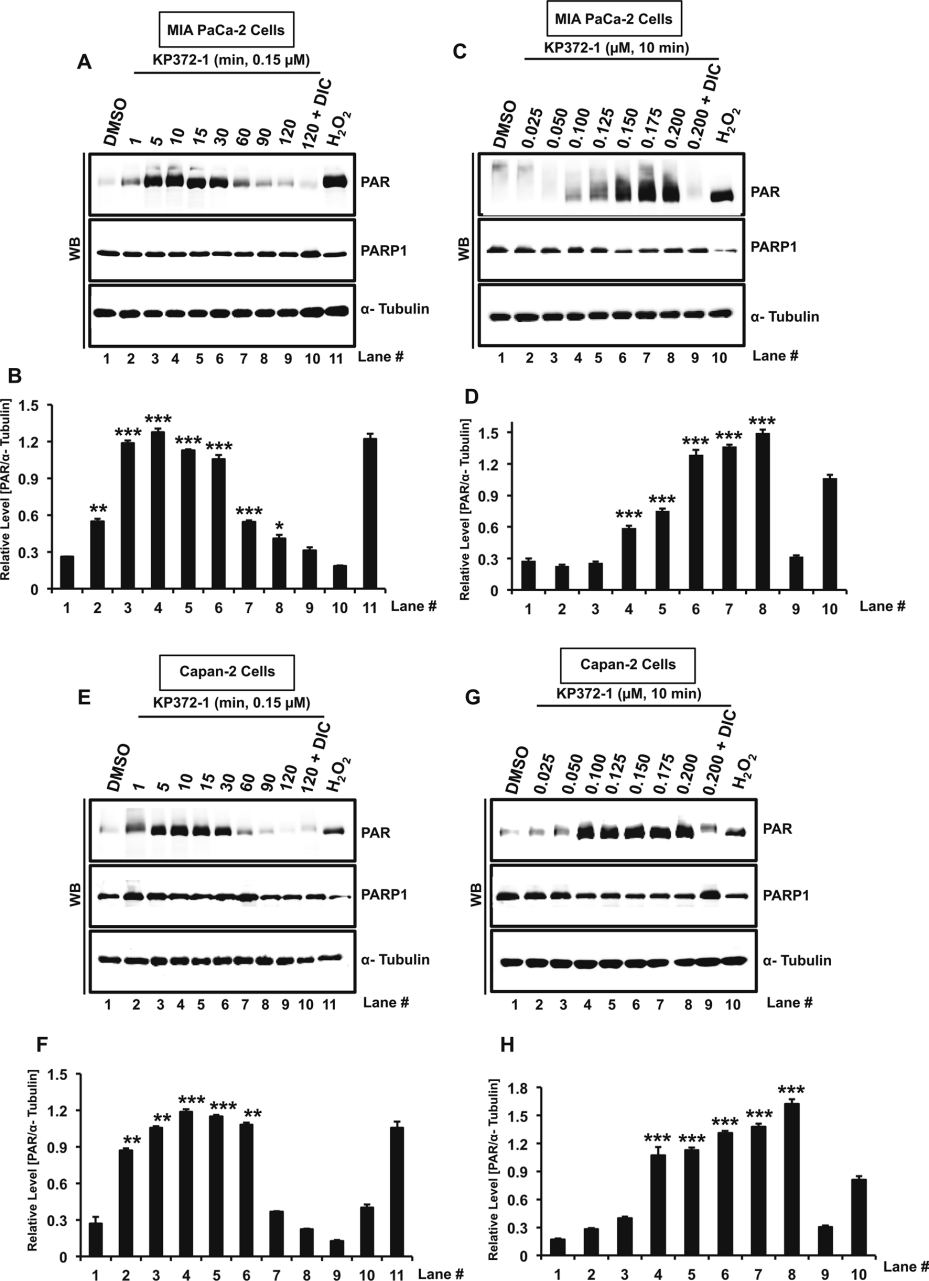
KP372-1 hyperactivates PARP1 in pancreatic cancer cells. (**A**–**H**) Assessment of PAR (poly-(ADP-ribose)) via Western blotting as a marker of PARP1 hyperactivation by KP372-1-induced DNA damage. MIA PaCa-2 cells treated with 0.15 µM KP372-1 ± 50 µM DIC for indicated time (min) points (**A**,**B**), blot image and quantification, respectively, and with indicated dose of KP372-1 (µM) ± 50 µM DIC for 10 min (**C**,**D**), blot image and quantification, respectively. Capan-2 cells treated with 0.15 µM KP372-1 ± 50 µM DIC for indicated time (min) points (**E**,**F**), blot image and quantification, respectively, and with indicated dose of KP372-1 (µM) ± 50 µM DIC for 10 min (**G**,**H**), blot image and quantification, respectively. Representative Western blot images are presented. Bar graphs represent quantification of band intensities (means ± SEM) of PAR normalized to α-tubulin of respective sample from n = 5. Band intensities were detected by ImageJ software (version 1.53c, https://imagej.net) and α-tubulin was used as loading control. Cell lysates prepared from H_2_O_2_-treated cells (1 mM, 15 min in 1× PBS) served as positive control. *p* values were obtained via two-tailed student’s t-tests. *p < 0.05; **p < 0.01; ***p < 0.001, comparing treated with control (DMSO) samples. WB; Western blot. Raw data for all the Western blot images are provided in the supplementary information.

Profound DNA damage emanating from massive ROS production in pancreatic cancer cells strongly indicated potential hyperactivation of PARP1 after KP372-1 treatment. To investigate this possibility, time-course and dose–response studies were carried out to assess poly(ADP-ribose) (PAR) formation using Western blotting as a measure of PARP1 hyperactivation. Cell lysate obtained from H_2_O_2_ treated cells were utilized as positive control. Dramatic elevation in PAR formation was observed at multiple time points within a 2 h window in MIA PaCa-2 cells treated with 0.15 µM KP372-1, where co-treatment with DIC brought down PAR formation similar to DMSO treated control cells (Fig. 6A,B). Next, we carried out dose–response experiments with 10 min KP372-1 treatment. We found a clear dose-dependent enhancement of PAR formation (Fig. 6C,D). To further validate PARP1 hyperactivation by KP372-1 treatment, similar studies were carried out in Capan-2 cells. As expected, we noticed a clear time- and dose-dependent induction of PAR formation in Capan-2 cells (Fig. 6E–H, respectively) similar to that of MIA PaCa-2 cells. NQO1-deficient PANC-1 cells treated with similar conditions did not exhibit increased PAR signal above background levels (Fig. S2E). The observed trend of PAR formation in our time course studies is likely due to the activation of PAR metabolizing enzyme poly(ADP-ribose) glycohydrolase (PARG). Moreover, the antibody we used in our study to evaluate PAR is specific to long chain PAR, which might be rapidly metabolized by PARG resulting in the observed trend. However, we do not rule out the sustained short chain PAR signal beyond time point used here. Nonetheless, these data clearly suggest that KP372-1 treatment induces PARP1 hyperactivation in NQO1-expressing pancreatic cancer cells.

### KP372-1 treatment activates caspase-3 in pancreatic cancer cells

**Figure 7:**
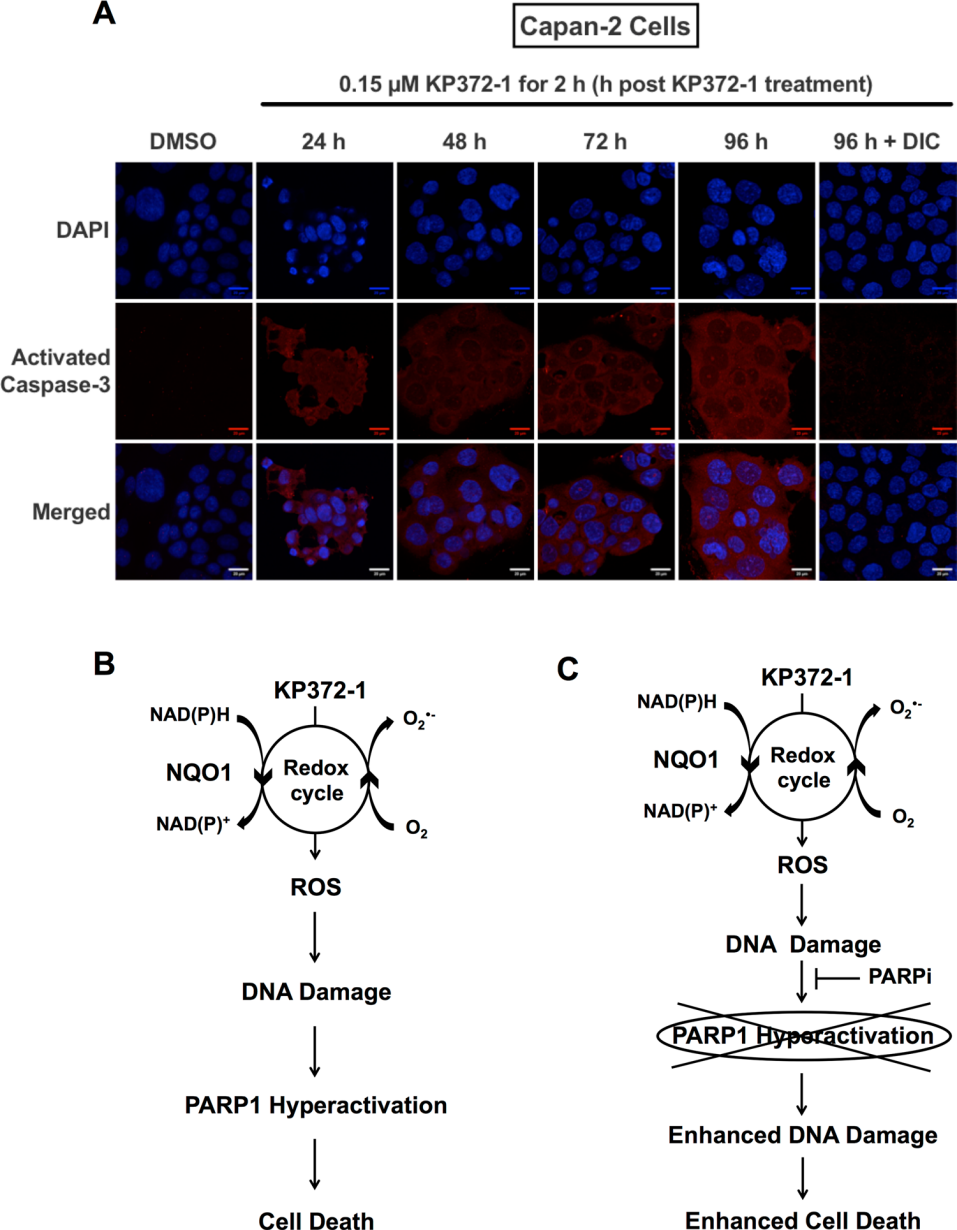
KP372-1 treatment activates caspase-3 in pancreatic cancer cells. (**A**) Evaluation of caspase activation via confocal immunofluorescence microscopy using cleaved caspase-3 antibody (i.e., activated caspase-3). Capan-2 cells were treated with 0.15 µM KP372-1 ± 50 µM DIC for 2 h and released for indicated time points (h) prior to processing for immunofluorescence. Note strong staining of capan-2 cells with activated caspase-3 from 24 to 96 h, whereas, DMSO treated cells do not show appreciable signal. Representative image from n = 4 biological repeats has shown. (**B**,**C**) Model providing mechanistic insight into cellular consequences of KP372-1 exposure to NQO1 overexpressing cancer cells.

To gain insight into cell death pathway instigated in pancreatic cancer by KP372-1 treatment, we investigated caspase activation. For evaluation of caspase activation, we employed immunofluorescence confocal microscopy using antibody specific to cleaved caspase-3 (i.e., activated caspase-3). Capan-2 cells were treated with 0.15 µM KP372-1 for 2 h and released for indicated time points prior to fixing, incubating with antibodies and image acquisition. Compared to control (DMSO-treated) cells, KP372-1 treated cells clearly showed enhanced signal for activated caspase-3 at all the time points within 24–96 h window (Fig. 7A). Importantly, DIC treatment blocks KP372-1-induced activation of caspase-3 (Fig. 7A). Collectively, these findings suggest that KP372-1 treatment leads to caspase-3 activation in pancreatic cancer cells to initiate cell death.

Based on data presented in Figs. 1, 2, 3, 4 and 5, we envisioned a model presented in Fig. 7B for KP372-1-induced cytotoxicity, where NQO1-dependent redox cycling generates ROS that cause DNA damage. To counteract DNA damage, cells hyperactivate PARP1 in an attempt to repair the damage. However, the amount of DNA damage at higher doses of KP372-1 exhausts the cellular DNA repair capacity, and cells activate caspase-3 to initiate cell death (Fig. 7B). Based on this model, we predict that inducing DNA damage with KP372-1 and simultaneously blocking PARP1 activity should lead to augmented DNA damage and cause enhanced cell death at lower doses of KP372-1 (Fig. 7C).

### Combination of KP372-1 and PARP inhibition enhances KP372-1-induced cytotoxicity in pancreatic cancer cells

**Figure 8:**
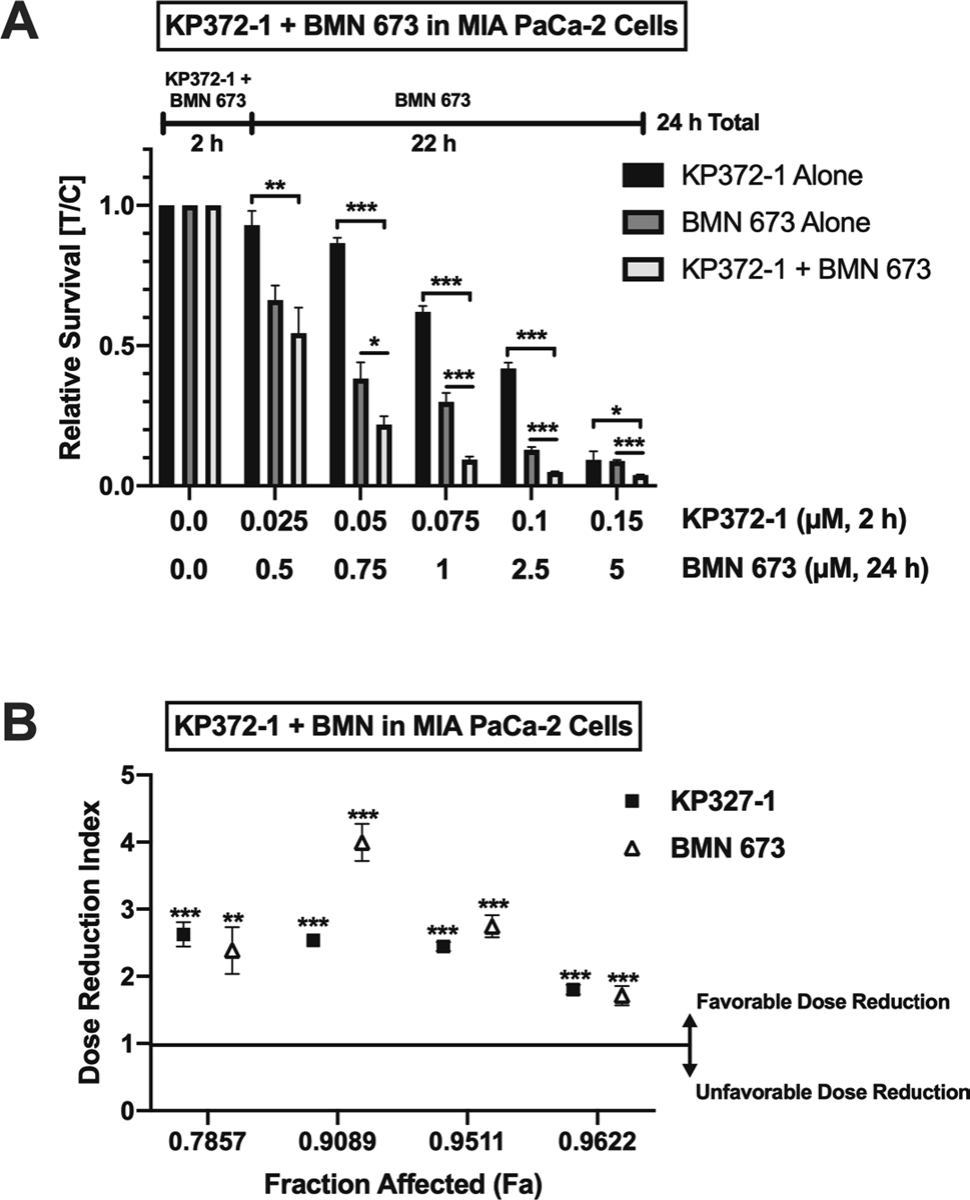
KP372-1 + PARP inhibition combination enhances cytotoxicity of pancreatic cancer cells. Relative survival measured by DNA content assay in the presence of indicated µM concentrations of KP372-1 ± BMN 673 (PARP1/2 inhibitor). (**A**) MIA PaCa-2 cells were treated with BMN 673 and KP372-1 in the order indicated by the outline above the graph. Bar graph represent means ± SEM for individual or combination treatment over control (i.e., DMSO) treated (T/C) samples from n = 3, each in triplicate. *p* values were obtained via two-tailed student’s t-tests. *p < 0.05; **p < 0.01; ***p < 0.001, comparing combination with individual treatments. (**B**) Dose reduction index (DRI) values were calculated using the survival data from panel A as input via CompuSyn 1.0 software (www.combosyn.com) where any value above 1 indicates a reduction in dose for individual agents to achieve the given fraction affected (Fa) upon combination. *p* values were obtained via two-tailed student’s t-tests. *p < 0.05; **p < 0.01; ***p < 0.001, comparing the dose reduction value to the additive value of 1.

Observed PARP1 hyperactivation in response to KP372-1 treatment (Fig. 6) suggested that pancreatic cancer cells rely on PARP1 activity to counteract cellular stress created by KP372-1. Therefore, we reasoned that blocking PARP1 activity in addition to KP372-1 treatment should further augment cytotoxicity of pancreatic cancer cells (Fig. 7C). To test this notion, we carried out cell survival studies with a potent FDA-approved PARP inhibitor, BMN 673 (talazoparib), in combination with KP372-1. As predicted, we observed significant enhancement of cytotoxicity of MIA PaCa-2 cells with multiple doses of KP372-1 + BMN 673 in combination compared to either of these agents alone (Fig. 8A). To gain further insight into the effectiveness of combination treatment, we utilized data presented in Fig. 8A as input and calculated the dose reduction index (DRI) values as a function of the fraction affected (Fa). The combination of KP372-1 and BMN 673 resulted in favorable dose reductions of both compounds, providing additional evidence that the combination treatment is more effective than single agents alone (Fig. 8B). For example, to achieve Fa = 0.9 in combination, only 0.075 µM KP327-1 and 1 µM BMN 673 is required (Fig. 8A), which correlates to a favorable DRI value of 2.53 for KP327-1 and 3.99 for BMN 673 (Fig. 8B). Finally, the combination index (CI) values and associated descriptions further support the effectiveness of KP372-1 + BMN 673 (Table S1). Collectively, these data suggest that combination of KP372-1 with the FDA-approved PARP inhibitor BMN 673 induces enhanced cell death in pancreatic cancer cells.

## Discussion

Pancreatic cancer remains a formidable challenge to overcome and efficient tumor-selective chemotherapeutic agents are critically needed. Conventional DNA damaging agents including gemcitabine, cisplatin, oxaliplatin, irinotecan, fluorouracil, and nab-paclitaxel have been explored as treatment options for pancreatic cancers. However, these agents lack tumor selectivity. Moreover, intrinsic or accumulated drug resistance rapidly makes these agents ineffective and severely limits the therapeutic window^33^. Elevated NQO1 overexpression in a variety of solid tumors, including pancreatic cancer, has emerged as an effective tumor-selective target^3–6^. Here, we show that KP372-1-induced robust DNA damage response ensuing hyperactivation of DNA damage sensor protein poly(ADP-ribose) polymerase 1 (PARP1) offers a promising targeted chemotherapeutic strategy against pancreatic cancer. In support of this major conclusion, we provide multiple lines of evidence including; (1) NQO1 expression is frequent in pancreatic cancers (Fig. 1), (2) NQO1-dependent redox cycling of KP372-1 selectively sensitizes pancreatic cancer cells (Fig. 2), (3) robust DNA damage is created due to ROS production afforded by NQO1-dependent redox cycling of KP372-1 (Figs. 3, 4 and 5), (4) KP372-1-induced DNA damage hyperactivates key damage sensor protein PARP1 leading to caspase-3 activation (Figs. 6 and 7), and (5) cytotoxicity of pancreatic cancer cells was further enhanced by simultaneously blocking PARP1 enzymatic activity through an FDA-approved inhibitor (Fig. 8). To our knowledge, this is the first study providing a mechanistic basis of KP372-1-induced cytotoxicity due to the induction of DNA damage and consequent PARP1 hyperactivation in pancreatic cancer cells.

Overexpression of NQO1 in solid cancers, including pancreatic cancer, is well-known; however, the actual mechanism driving high NQO1 expression remains elusive. NQO1 is a prototypical target gene for a transcription factor, nuclear factor erythroid 2 p45-related factor 2 (Nrf2)^34,35^. In response to increased oxidative stress, Nrf2 binds to a DNA sequence known as the “antioxidant response element” (ARE) to upregulate downstream target genes, including NQO1^34–40^. Some studies suggest that high levels of oxidative stress and inflammation in cancer cell cells activate NF-κB signaling via inhibiting p53 to drive NQO1 expression during carcinogenesis^41–43^. It is also reported that NQO1 elevation is a consequence of mutant K-RAS-driven Nrf2 overexpression in cancer cells^44^. Regardless of the exact mechanism, recurrent NQO1 overexpression and concomitant down-regulation of Catalase leading to high NQO1/Catalase ratios have been demonstrated as a promising therapeutic strategy against pancreatic cancers^4–6,45^. This study further demonstrates high levels of NQO1 expression in patient sample data obtained from Oncomine as well as from some of the commonly utilized pancreatic cancer cell lines (Fig. 1). Importantly, here we show that high NQO1 expression exclusively sensitizes a variety of pancreatic cancer cells to a novel NQO1 redox cycling agent KP372-1 (Fig. 2).

Initial studies reported KP372-1 as an inhibitor of PDK1/Akt signaling pathways compromising cell proliferation and promoting apoptosis^18–21^. However, Zhao and colleagues challenged the notion of KP372-1 being an inhibitor of PDK1/Akt signaling and found that KP372-1 actually enhances Akt phosphorylation^17^. Consistent with Zhao and colleagues’ findings, we also observed transiently increased Akt phosphorylation in pancreatic cancer cells treated with KP372-1 (Fig. S3). However, Akt phosphorylation induced by KP372-1 treatment appears to be independent of NQO1 status, since Akt phosphorylation remains intact in MIA PaCa-2 and Capan-2 cells treated with KP372-1 after siRNA-mediated knockdown of NQO1 (Fig. S3). Also, despite exhibiting strong Akt phosphorylation comparable to that of MIA PaCa-2 and Capan-2 (S3), naturally NQO1-deficient PANC-1 cells (Fig. 1) showed no toxicity against KP372-1 (Figs. 2, S1, S2 and S3). Furthermore, NQO1-depleted MIA PaCa-2 showed no sensitivity to KP372-1 despite maintaining the intact Akt-phosphorylation, (Figs. 2 and S3). Collectively, these observations indicate that cytotoxicity instigated by KP372-1 is independent of Akt phosphorylation.

The mechanism of cytotoxicity instigated by KP372-1 in pancreatic cancer cells illustrated here (Figs. 1, 2, 3, 4, 5, 6 and 7) defines the therapeutic potential of this compound. The redox cycling of KP372-1 causes dramatic elevation in ROS formation (Fig. 3), which is consistent with a previous report^17^. Here, we demonstrate for the first time that ROS generated by KP372-1 is sufficient to instigate extensive DNA damage (Figs. 4 and 5). Induction of DNA damage by KP372-1 is similar to damage created by redox cycling of another NQO1 substrate, β-lapachone (β-lap) observed in several studies^5,6,45^. This is also the first report showing that DNA damage induced by KP372-1 hyperactivates the central damage sensor protein PARP1 ensuing dramatic increase in PAR (poly(ADP-ribose)) formation (Fig. 6). KP372-1-induced PARP1 hyperactivation observed here is similar to that of β-lap treated pancreatic cancer cells^4^. DNA damage induced caspase-3 activation by KP372-1 observed in this study in pancreatic cancer cells (Fig. 7) is consistent with a previous study reporting caspase-3 activation in other cancer cells^17^. Compared to KP372-1, β-lap suffers from several barriers including modest pharmacokinetics, limited bioavailability, poor aqueous solubility, short half-life, and necessity for a drug delivery system. Importantly, other major challenges with β-lap as monotherapy or in combination with other agents include dose-limiting anemia and methemoglobinemia^14–16^. KP372-1 appears to be a better drug candidate than β-lap because its potency is one magnitude higher than β-lap observed in this study (Figs. 2 and S1) and reported by others^17^. Also, KP372-1 (1) is compatible with oral administration, (2) has a large volume of distribution, (3) readily enters in the tumor, (4) exhibits long half-life, (5) has good bioavailability, and (6) mice can tolerate doses that produce significant anti-tumor activity without obvious toxicity^17^.

To counteract cytotoxicity and DNA damage induced by KP372-1, pancreatic cancer cells seem to rely on PARP1 activity for orchestrating DNA repair response (Fig. 6). This finding suggested that KP372-1 treatment with simultaneous inhibition of PARP could create a more potent cytotoxic response. Furthermore, β-lap + PARP inhibition (PARPi) clearly showed a synergistic cancer cell death response^6^. Finally, PARP inhibitors are effective against pancreatic cancer as a monotherapy or in combination with other agents^46–50^. These observations prompted us to test the effectiveness of the combination of KP372-1 with PARPi. Indeed, we observed enhanced cell death upon combining these two agents (Fig. 8). Collectively, KP372-1 demonstrates high potency as a single agent or in combination with PARPi against pancreatic cancer cells. In principle, KP372-1 can be combined with all the agents that showed synergy/enhanced cytotoxicity with β-lap^5,6,15,45^. Also, the mechanism of KP372-1-induced cytotoxicity delineated here for NQO1-overexpressing pancreatic cancer cells is likely to be similar for other NQO1+ solid cancers of lung, colon, breast, prostate and other tissues. Dose optimization for KP372-1 treatments alone and/or in combination with agents utilized in aforementioned studies are currently being evaluated in our laboratory against various cell line models for listed cancers to show a broad applicability of KP372-1 against other cancers.

In summary, KP372-1 sensitizes numerous NQO1-expressing pancreatic cancer cells, and spares immortalized normal pancreatic duct cells, hTERT-HPNE. Notably, we found that KP372-1 is at least ~ 10- to 20-fold more potent than another NQO1 substrate, β-lap (ARQ761, under phase 1 clinical trials against pancreatic cancers, NCT02514031). Importantly, at the mechanistic level, our data strongly suggest that reactive oxygen species produced by NQO1-dependent redox cycling of KP372-1 cause massive DNA damage in pancreatic cancer cells within two hours of treatment. Furthermore, we found that KP372-1-induced DNA damage hyperactivates the central DNA damage sensor protein PARP1 and causes caspase-3 activation to promote cell death. Our data also show that the combination of KP372-1 with PARP inhibition creates enhanced sensitivity in pancreatic cancer cells. Collectively, our mechanistic study lays an essential foundation to establish KP372-1 as a promising chemotherapeutic agent against pancreatic and other NQO1-overexpressing cancers.

## Supplementary information

Supplementary Information.

## Abbreviations

DIC: Dicoumarol
β-lap: β-Lapachone
NAC: N-Acetylcysteine amide
NQO1: NAD(P)H:quinone oxidoreductase 1
PARP1: Poly(ADP-ribose) polymerase 1
PARPi: PARP inhibition
ROS: Reactive oxygen species
