# Supplementary material for: DNA damage induced by KP372-1 hyperactivates PARP1 and enhances lethality of pancreatic cancer cells with PARP inhibition
Source: Sci Rep. 2020 Nov 19;10:20210. doi: 10.1038/s41598-020-76850-4 (PMC7677541; doi:10.1038/s41598-020-76850-4)

DNA damage induced by KP372-1 hyperactivates PARP1 and enhances lethality of pancreatic cancer cells with PARP inhibition

Talysa Viera† and Praveen L. Patidar† *

Department of Chemistry, New Mexico Institute of Mining and Technology, Socorro, NM, 87801, USA.

† Equal first author contribution

* To whom correspondence should be addressed: Praveen L. Patidar, Department of Chemistry, New Mexico Institute of Mining and Technology, 801 Leroy Pl., Socorro, NM, 87801, USA; *Praveen.Patidar@nmt.edu;* Phone: +1 575 835 5007, Fax: +1 575 835 5364

Supplementary Material

Table S1. Combination index values for MIA PaCa-2 cells treated with KP372-1 and BMN 673. The combination index values (CI) for MIA PaCa-2 cells treated with KP372-1 and BMN 673 in Figure 8A displayed as CI ± S.D. with accompanying synergy description as defined by T.C. Chou [1]. All values display synergy to nearly additive combination index values. The strongest synergy is observed at a dose of 0.075 μM KP372-1 in combination with 1 μM BMN 673.

KP372-1 Dose (μM)	BMN 673 Dose (μM)	Fraction affected (Fa)	Combination Index (CI)	Synergy Description[1]
0.025	0.5	0.5224 ± 0.03	1.061 ± 0.09	Nearly additive
0.05	0.75	0.7857 ± 0.03	0.808 ± 0.09	Moderate synergism
0.075	1	0.9089 ± 0.007	0.646 ± 0.03	Synergism
0.1	2.5	0.9511 ± 0.003	0.775 ± 0.03	Moderate synergism

Figure Legends

Figure S1. Sensitivity of β-lapachone (β-lap) against pancreatic cancer cells. (A-C) Relative survival measured by MTT assay in the presence of indicated μM concentrations of KP372-1 ± 50 μM dicoumarol (DIC, NQO1 inhibitor), for 2 h. Phenylarsine oxide (PAO) was used as a positive control. Graphs represent %means ± S.D. for KP372-1 or DIC or KP372-1 + DIC treated over control (i.e., DMSO) treated (T/C) samples for MIA PaCa-2 (A), Capan-2 (B), and PANC-1 cells (C) from n=4, each in triplicate. *p* values were obtained via an ordinary one-way ANOVA using the Dunnett's multiple comparisons test. **, $p<0.01$; ***, $p<0.001$; ****, p<0.0001; ns, not significant, comparing indicated drug treatments to the DMSO control. **(D-E)** Relative survival measured by DNA content assay in the presence of indicated μM concentrations of β-lap ± 50 μM dicoumarol (DIC, NQO1 inhibitor), for 2 h. Graphs represent means ± SEM for β-lap treated over control (i.e., DMSO) treated (T/C) samples for MIA PaCa-2 (A), Capan-2 (B) cells from n=4, each in triplicate. *p* values were obtained via two-tailed student's t-tests. *, $p<0.05$; **, $p<0.01$; ***, $p<0.001$, comparing β-lap with β-lap + DIC.

Figure S2. PANC-1 Cells are resistant to KP372-1 treatment. (A) Clonogenic survival in the presence of indicated concentrations (μM) of KP372-1 ± DIC for 2 h. **(B)** Relative levels of H_2O_2 production in control (DMSO), KP372-1 and KP372-1 ± N-acetylcysteine amide (NAC, 1 mM or 5 mM for total of 5 h (pre-treatment for 3 h and co-treatment for 2 h)) treated PANC-1 cells. **(C-D)** Relative levels of nuclear 8-oxoG signal in control (DMSO), 0.15 μM KP372-1, or 0.15 μM KP372-1 ± 50 μM DIC treated for 1 h were measured by immunofluorescence confocal microscopy. Cells treated with H_2O_2 (1 mM, 15 min in 1X PBS) served as positive control. Representative images of PANC-1 cells (C), and quantification of fluorescence signal (D). The scale bar represents 10 μm. Graphs represent the means (red bar) for treated/control samples from n=3, each performed in duplicate for total of 150 cells. *p* values were obtained via an

ordinary one-way ANOVA using the Dunnett's multiple comparisons test. ****, $p<0.0001$; ns, not significant, comparing indicated drug treatments to the DMSO control. **(E)** Assessment of phosphorylated H2AX (γH2AX) and PAR (poly-(ADP-ribose)) via Western blotting as a marker of DNA damage response induced by KP372-1. MIA PaCa-2 cells treated with 0.15 μM KP372-1 ± 50 μM DIC for indicated time (min) points.

Figure S3. KP372-1 promotes Akt phosphorylation independent of NQO1 expression. Assessment of phosphorylated Akt (pAkt) via Western blotting induced by KP372-1. **(A)** MIA PaCa-2 cells treated with 0.15 μM KP372-1 for indicated time (min) points. **(B)** Capan-2 cells treated with 0.15 μM KP372-1 for indicated time (min) points. **(C)** PANC-1 cells treated with 0.15 μM KP372-1 for indicated time (min) points. **(D)** MIA PaCa-2 cells + siSCR or siNQO1 treated with 0.15 μM KP372-1 for 15 min. **(E)** Capan-2 cells + siSCR or siNQO1 treated with 0.15 μM KP372-1 for 15 min. Representative Western blot images are shown here from n=4.

Figure S1

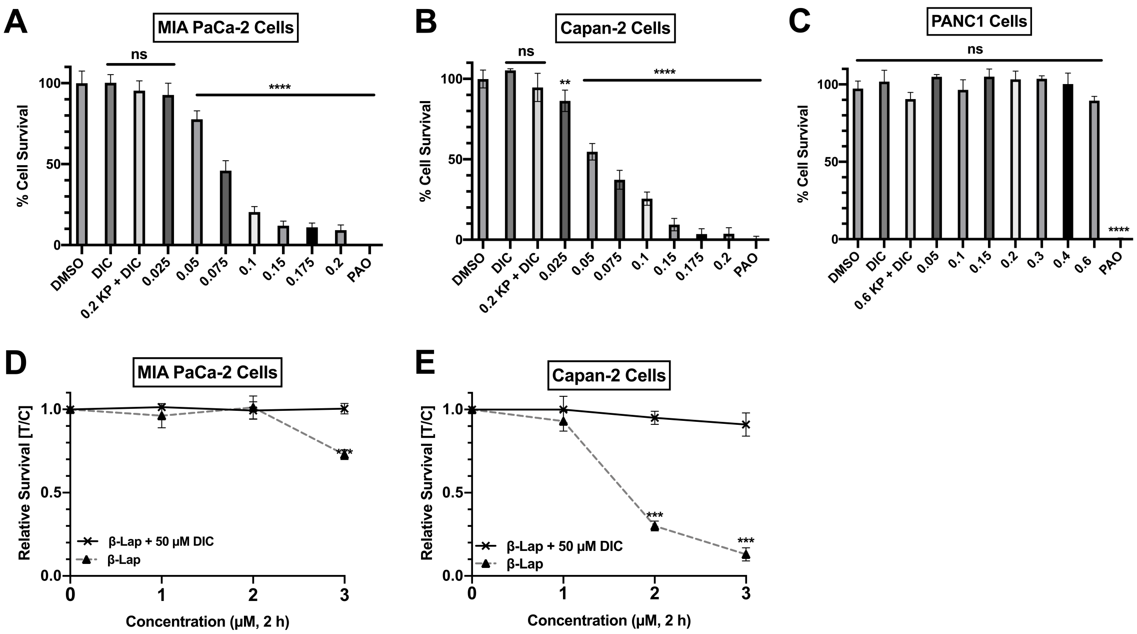

A
MIA PaCa-2 Cells
% Cell Survival
DMSO
DIC
0.2 KP + DIC
PAO
B
Capan-2 Cells
C
PANC1 Cells
0.6 KP + DIC
D
MIA PaCa-2 Cells
Relative Survival [T/C]
β-Lap + 50 µM DIC
β-Lap
Concentration (µM, 2 h)
E
Capan-2 Cells

Figure S2

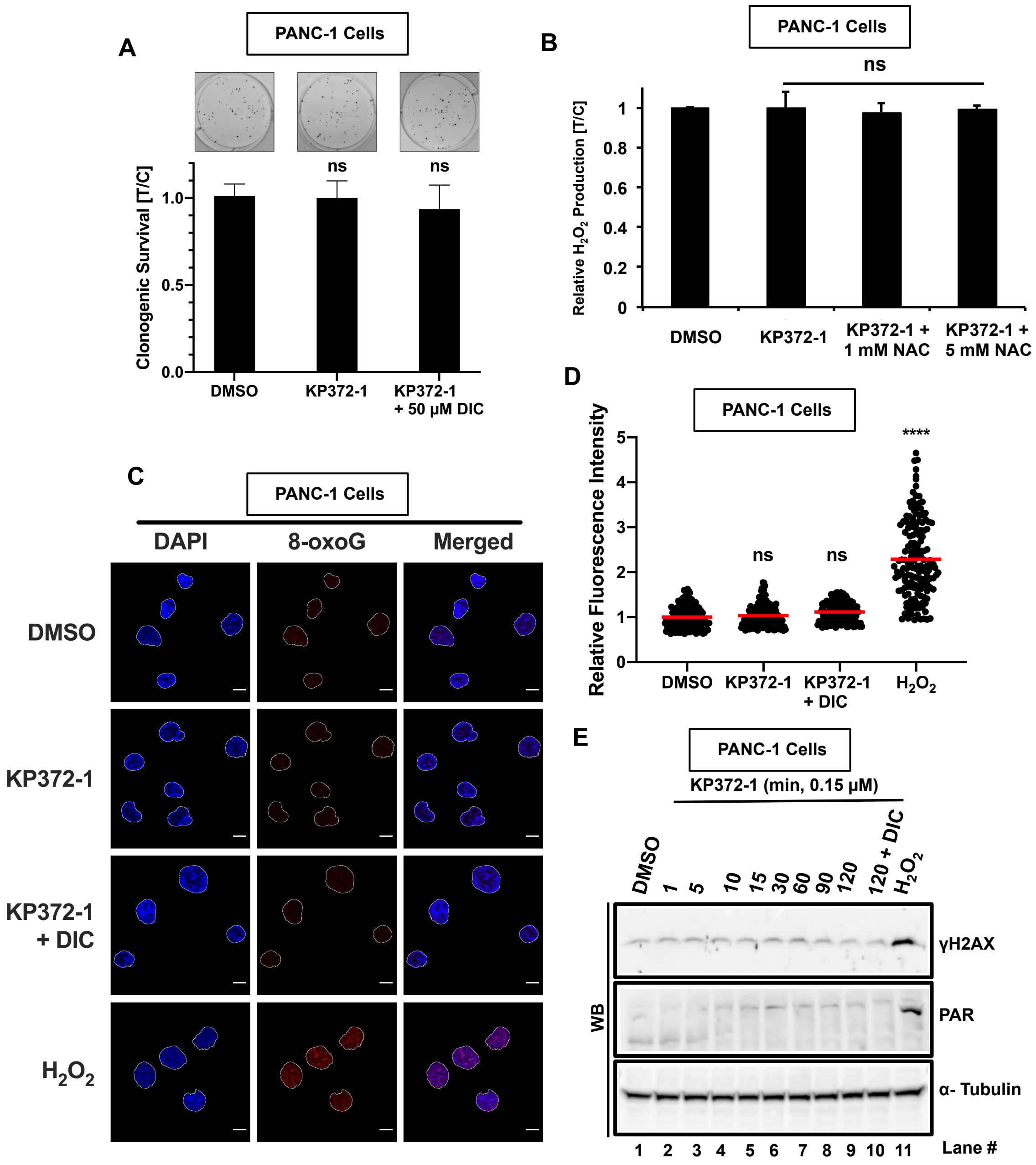

A
PANC-1 Cells
Clonogenic Survival [T/C]
1.0
0.5
0.0
ns
ns
DMSO
KP372-1
KP372-1
+ 50 μM DIC
B
PANC-1 Cells
ns
Relative H2O2 Production [T/C]
1
0.8
0.6
0.4
0.2
0
DMSO
KP372-1
KP372-1 +
1 mM NAC
KP372-1 +
5 mM NAC
C
PANC-1 Cells
DAPI
8-oxoG
Merged
DMSO
KP372-1
KP372-1
+ DIC
H2O2
D
PANC-1 Cells
Relative Fluorescence Intensity
5
4
3
2
1
0
****
ns
ns
DMSO
KP372-1
KP372-1
+ DIC
H2O2
E
PANC-1 Cells
KP372-1 (min, 0.15 μM)
DMSO
1
5
10
15
30
60
90
120
120 + DIC
H2O2
WB
γH2AX
PAR
α- Tubulin
1 2 3 4 5 6 7 8 9 10 11
Lane #

Figure S3

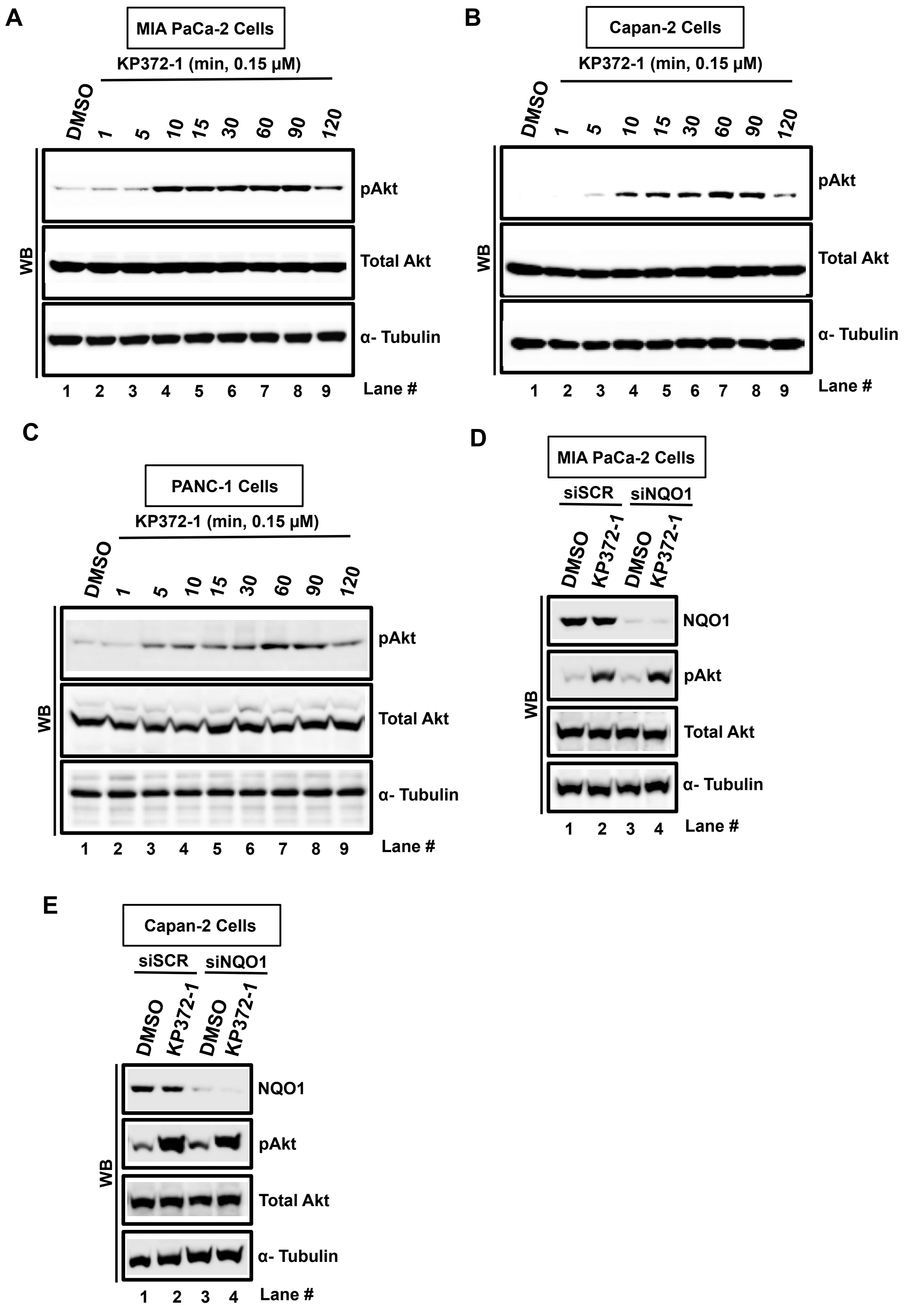
A
MIA PaCa-2 Cells
KP372-1 (min, 0.15 μM)
DMSO 1 5 10 15 30 60 90 120
pAkt
Total Akt
α- Tubulin
WB
1 2 3 4 5 6 7 8 9 Lane #
B
Capan-2 Cells
KP372-1 (min, 0.15 μM)
DMSO 1 5 10 15 30 60 90 120
pAkt
Total Akt
α- Tubulin
WB
1 2 3 4 5 6 7 8 9 Lane #
C
PANC-1 Cells
KP372-1 (min, 0.15 μM)
DMSO 1 5 10 15 30 60 90 120
pAkt
Total Akt
α- Tubulin
WB
1 2 3 4 5 6 7 8 9 Lane #
D
MIA PaCa-2 Cells
siSCR siNQO1
DMSO KP372-1 DMSO KP372-1
NQO1
pAkt
Total Akt
α- Tubulin
WB
1 2 3 4 Lane #
E
Capan-2 Cells
siSCR siNQO1
DMSO KP372-1 DMSO KP372-1
NQO1
pAkt
Total Akt
α- Tubulin
WB
1 2 3 4 Lane #

References

1 Chou, T. C. Preclinical versus clinical drug combination studies. *Leuk Lymphoma* **49**, 2059-2080, doi:10.1080/10428190802353591 (2008).

Figure 1F Supplementary

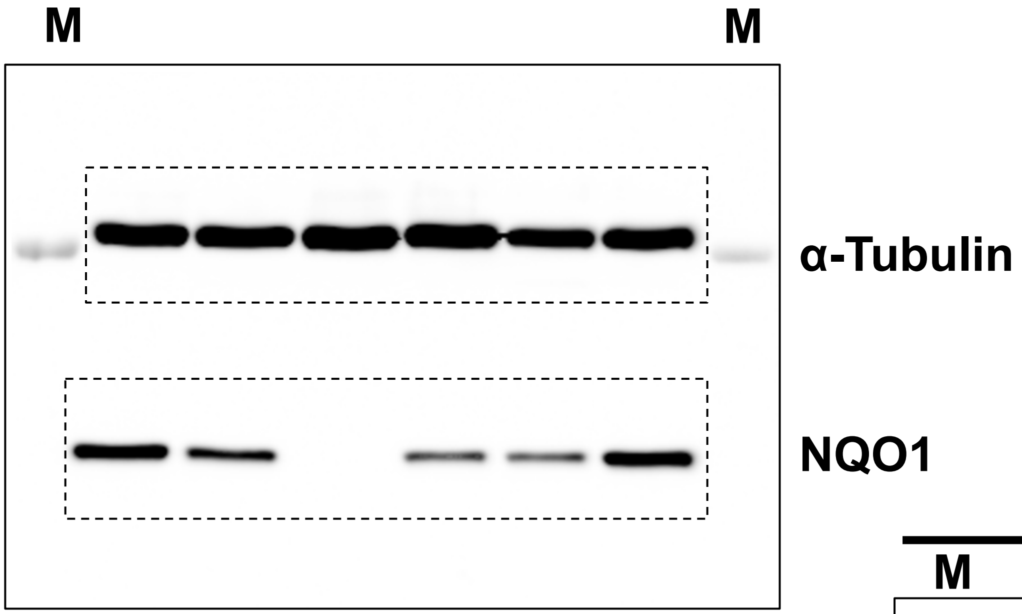

Lighter Exposure

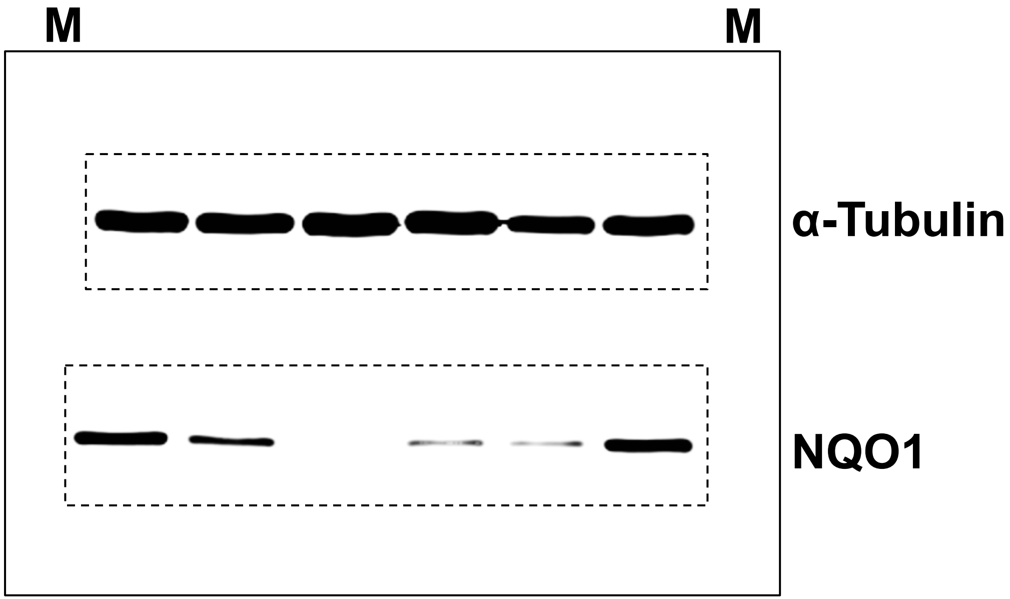

M: Molecular Weight Marker

Figure 2C Supplementary

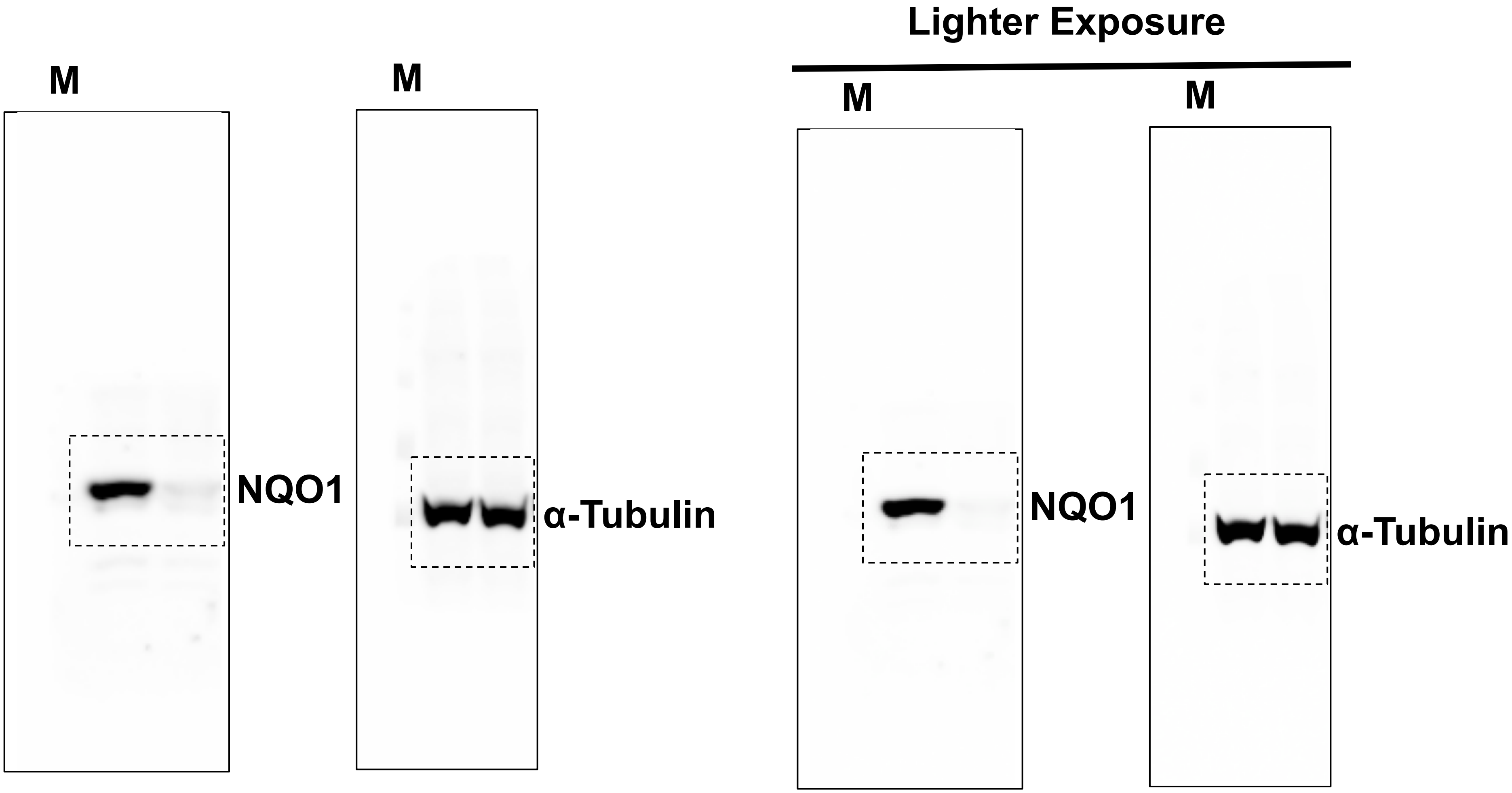

M: Molecular Weight Marker

Figure 5A Supplementary

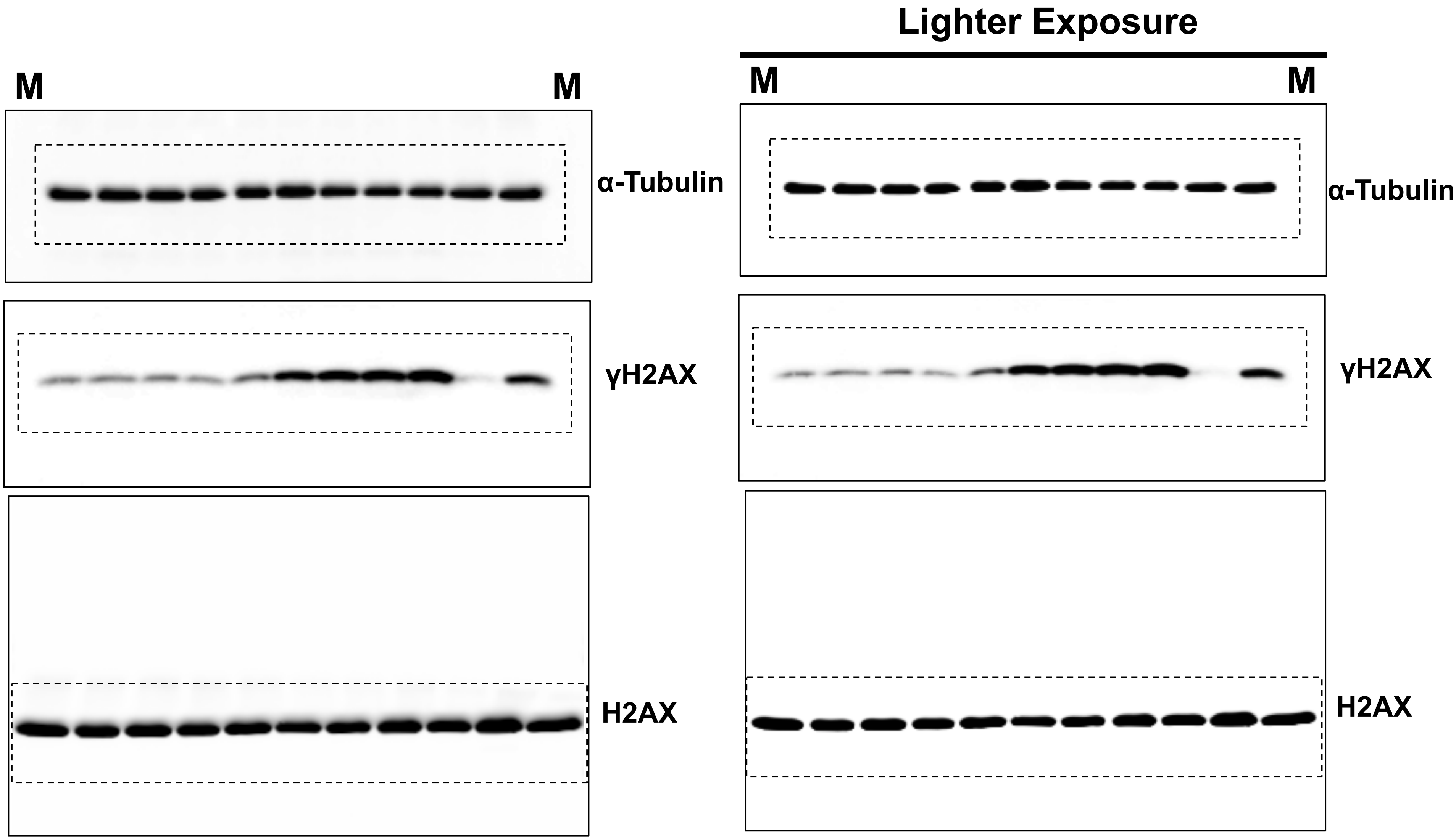

M: Molecular Weight Marker

Figure 5C Supplementary

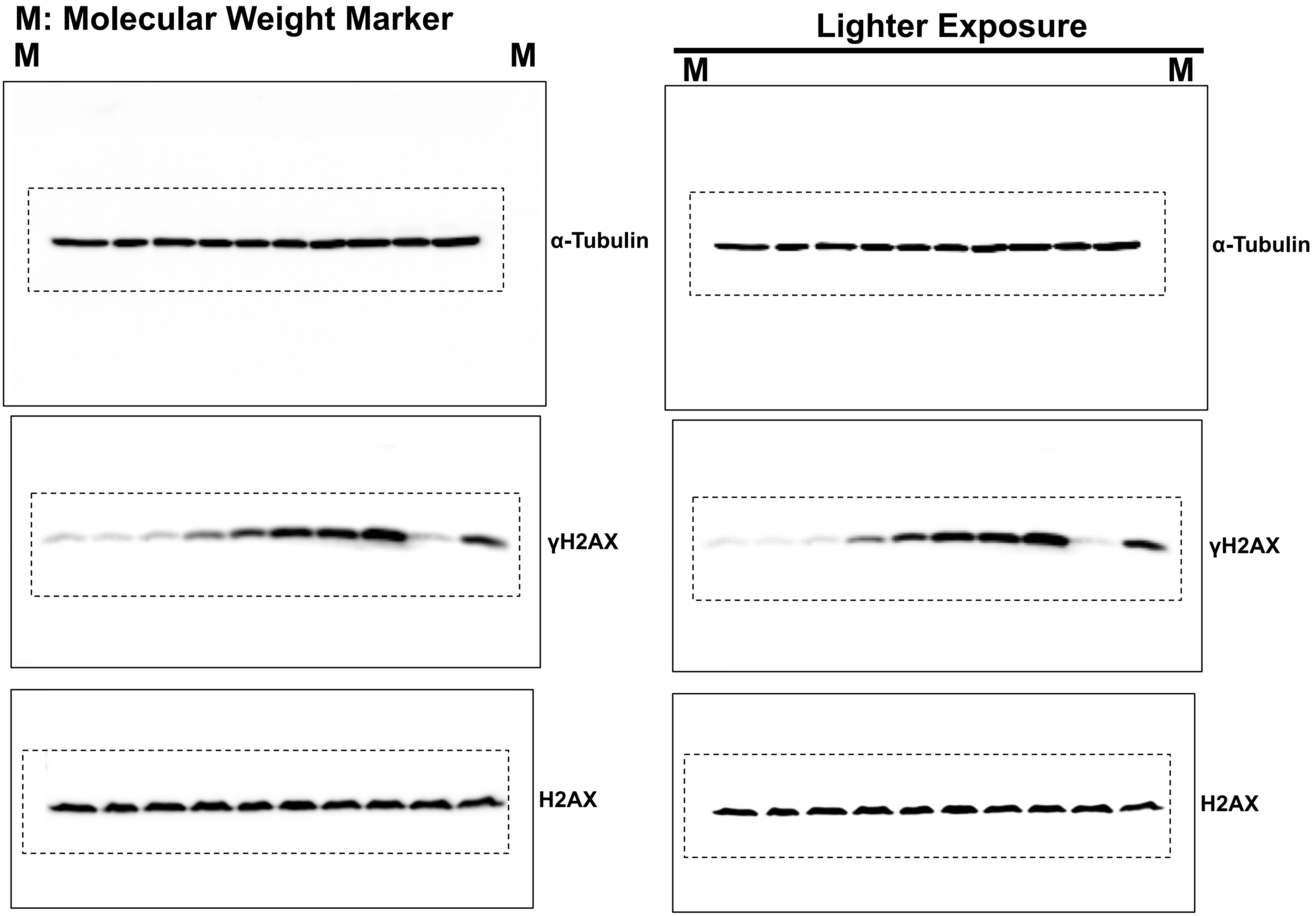

Figure 5E Supplementary

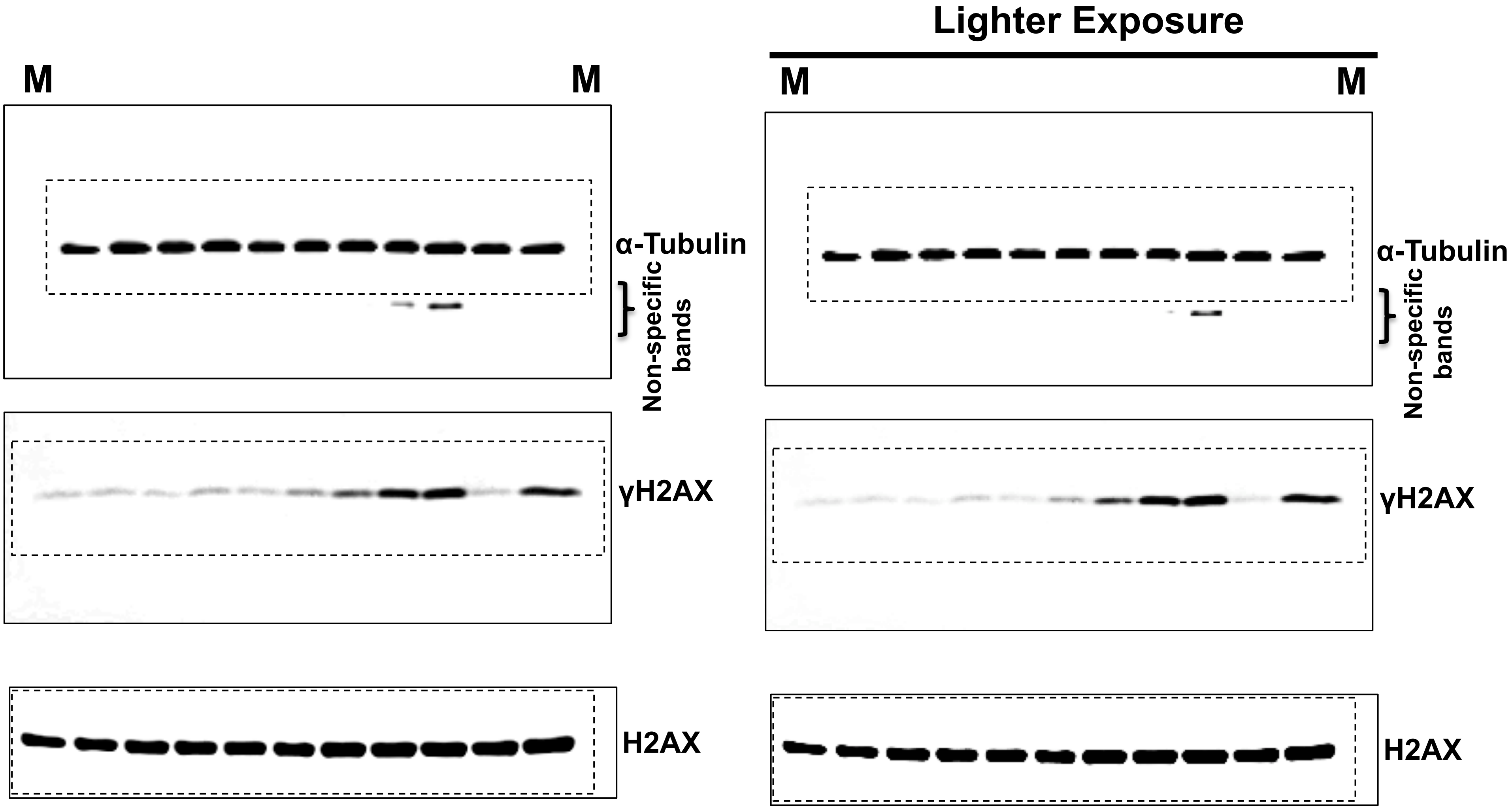

M: Molecular Weight Marker

Figure 5G Supplementary

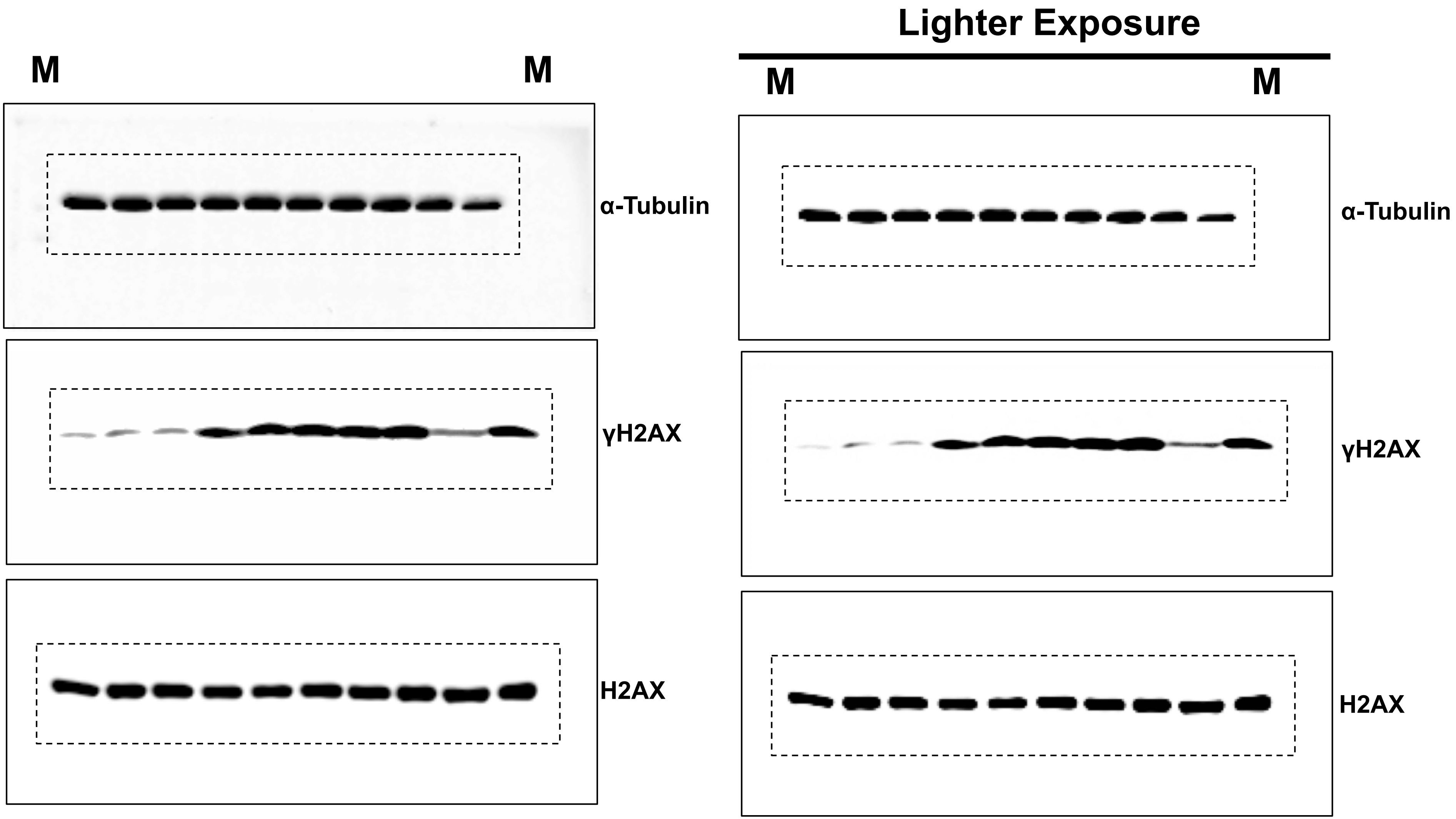

M: Molecular Weight Marker

Figure 6A Supplementary

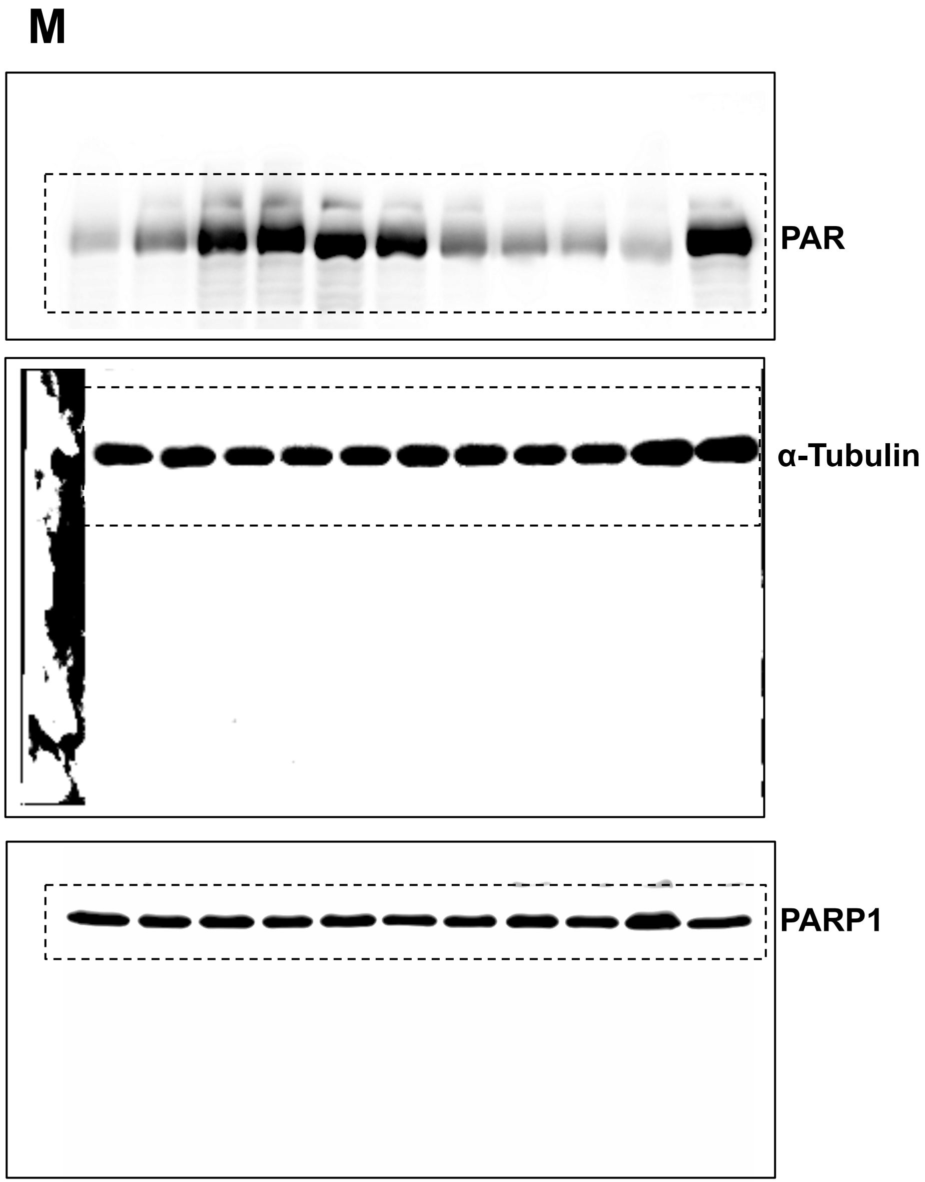

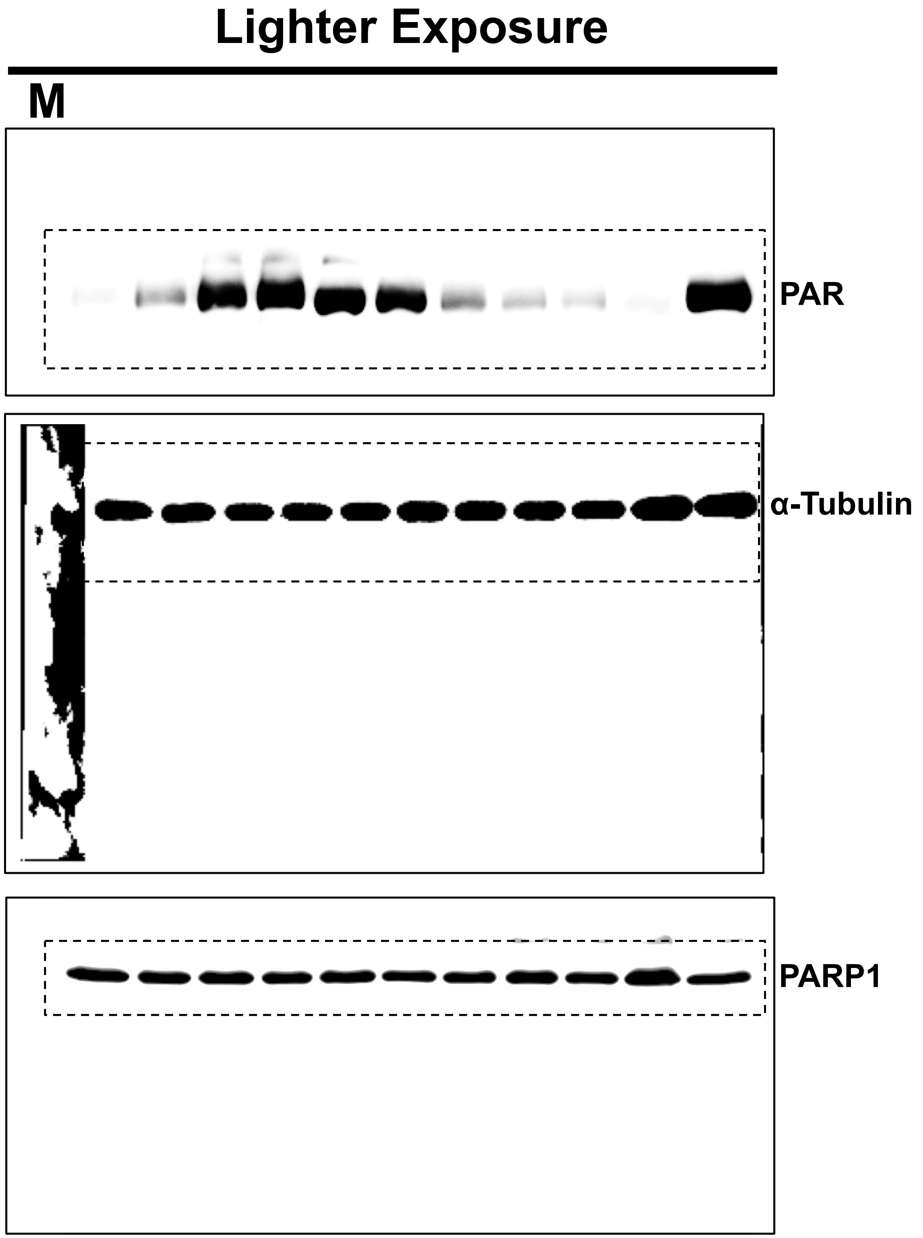

M: Molecular Weight Marker

Figure 6C Supplementary

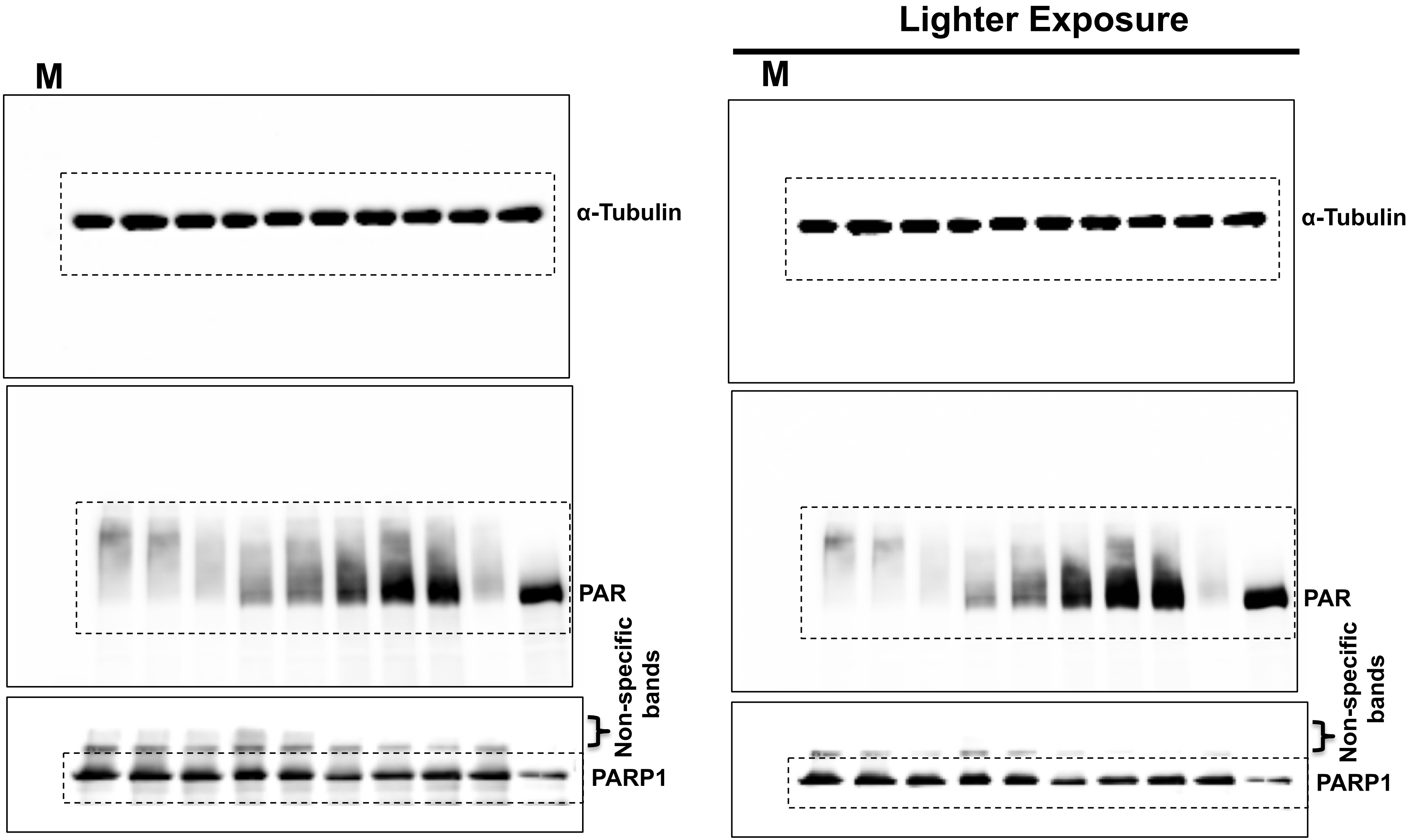

M: Molecular Weight Marker

Figure 6E Supplementary

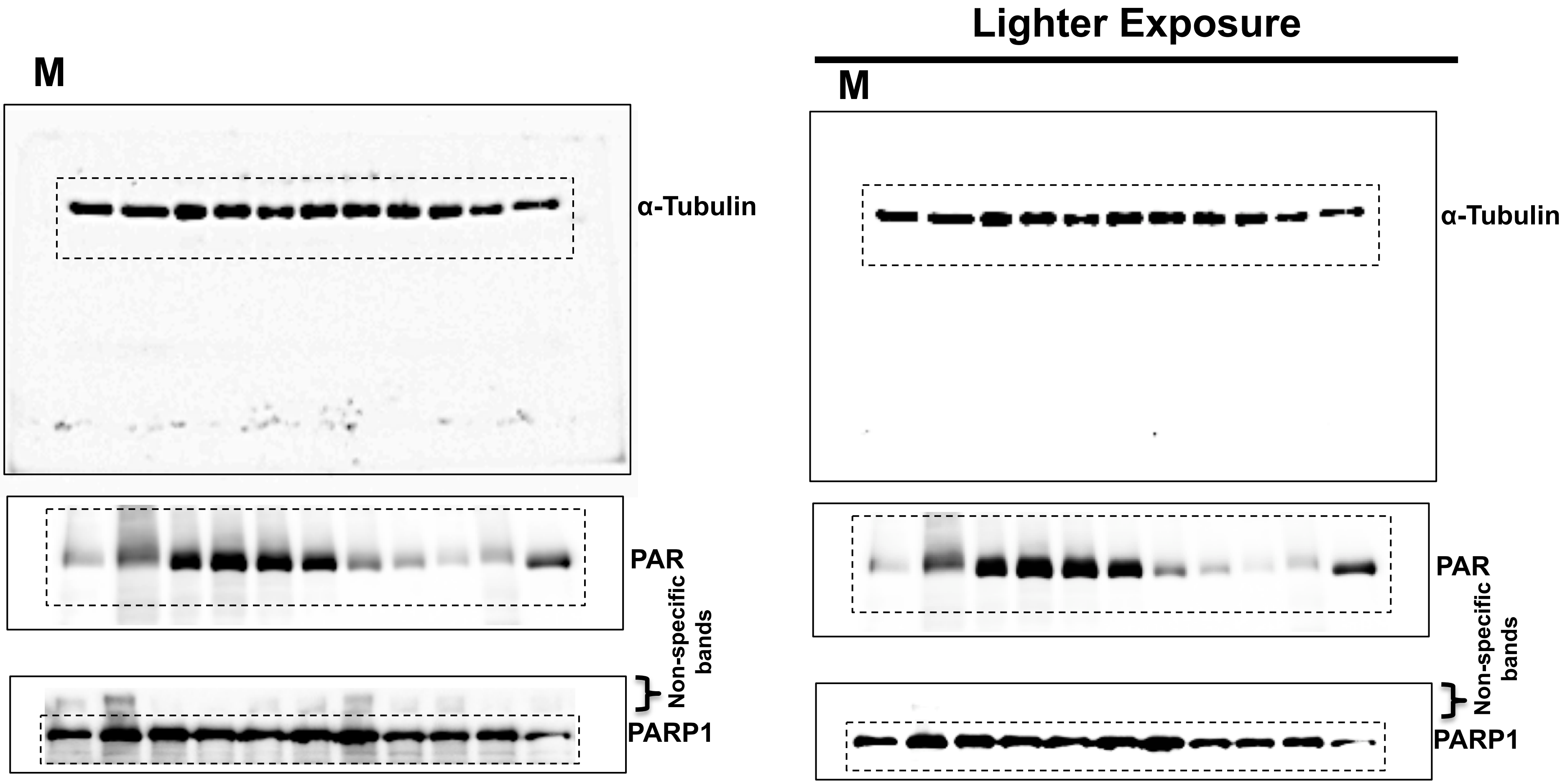

M: Molecular Weight Marker

Figure 6G Supplementary

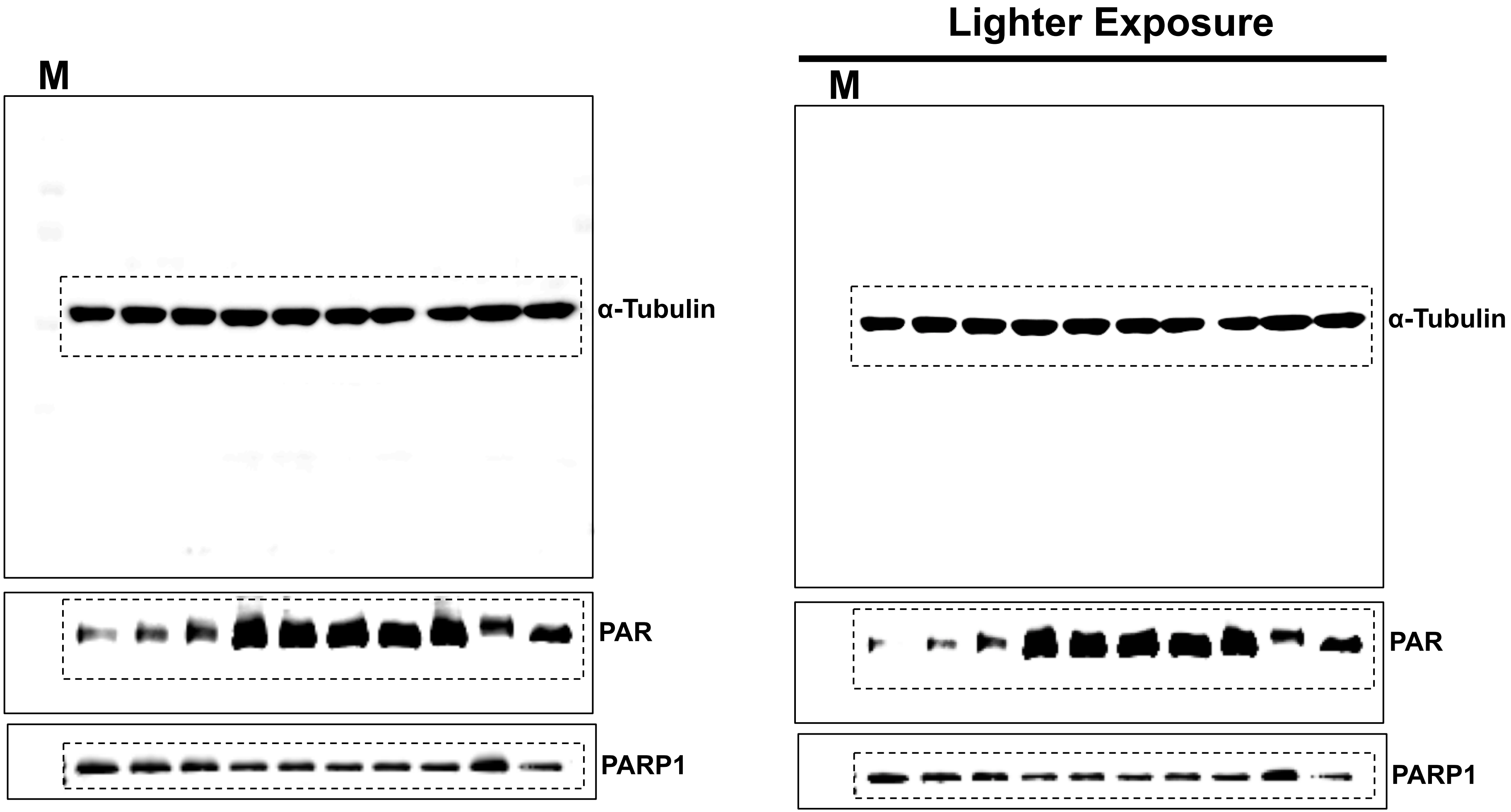

M: Molecular Weight Marker

Figure S2E Supplementary

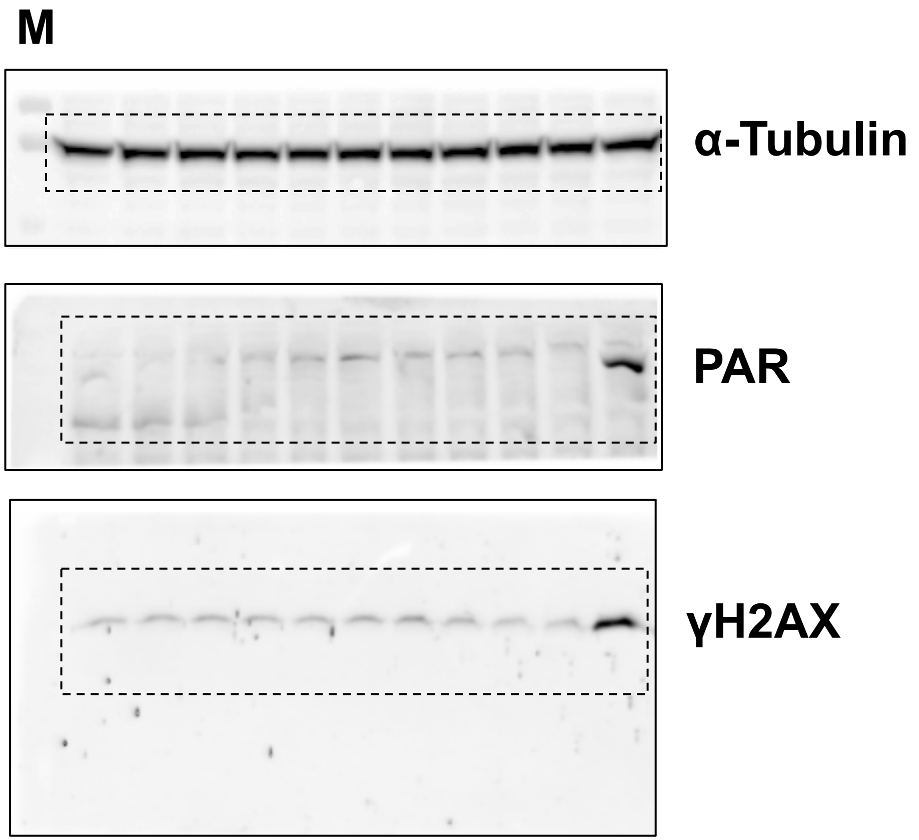

M: Molecular Weight Marker

Figure S3A Supplementary

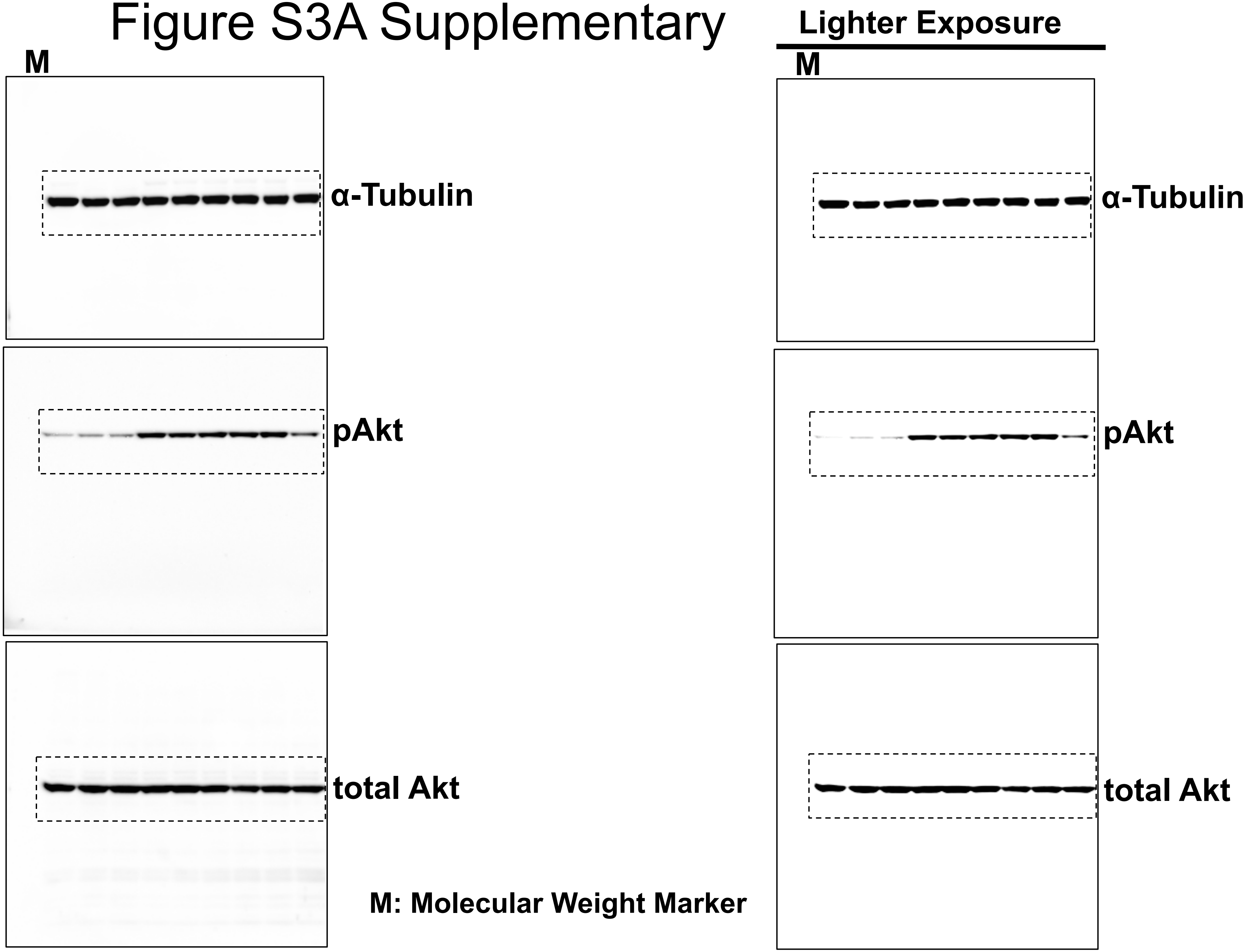

M: Molecular Weight Marker

Figure S3B Supplementary

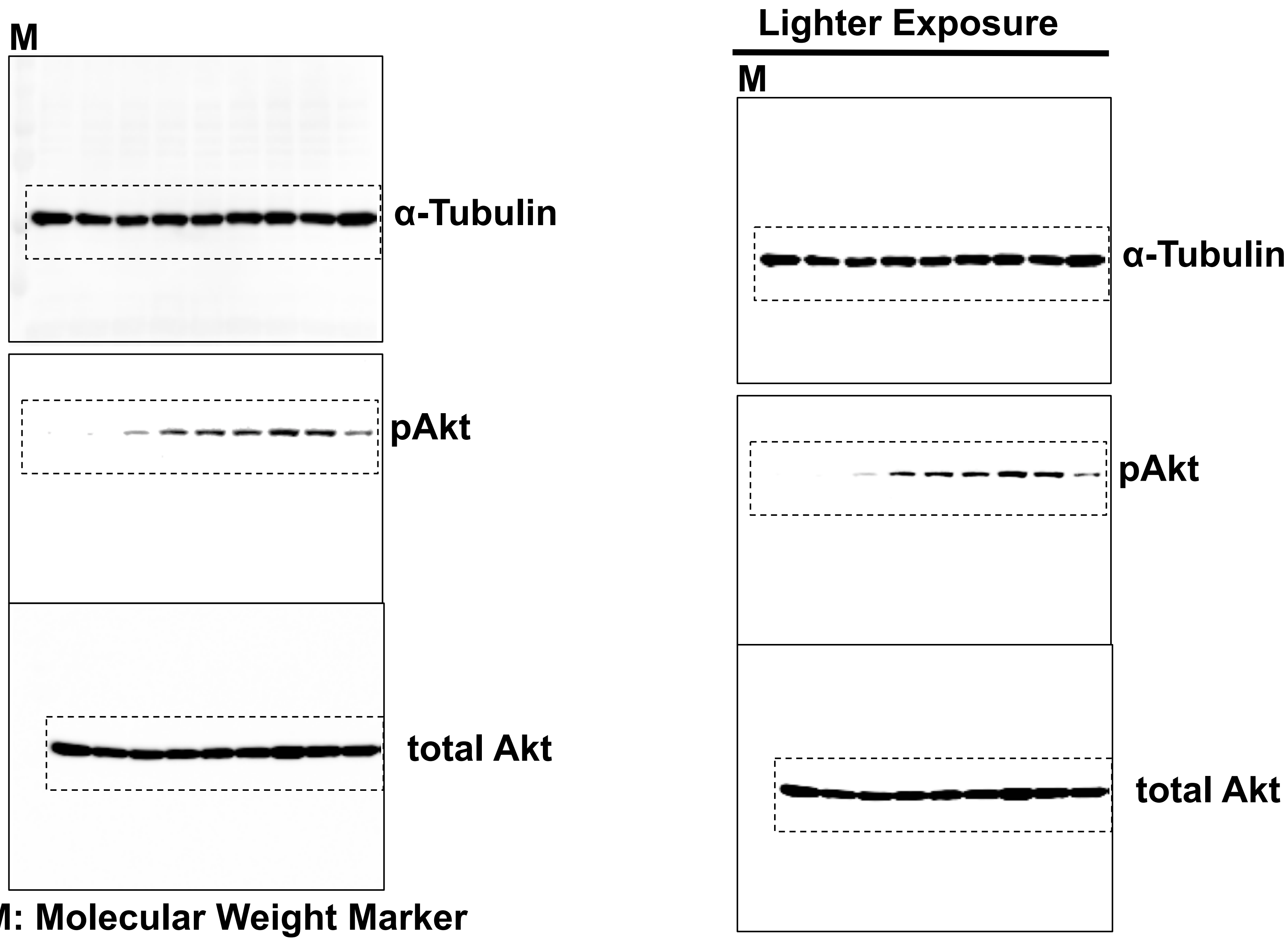

M: Molecular Weight Marker

Figure S3C Supplementary

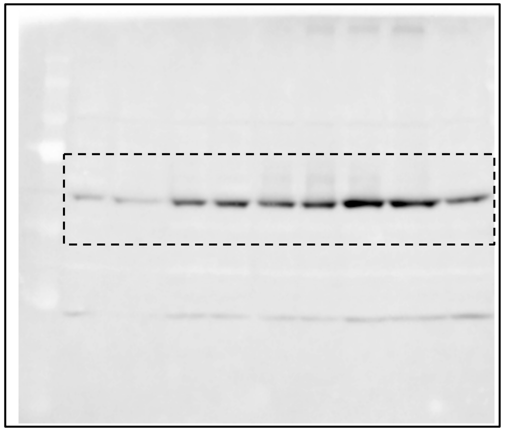

pAkt

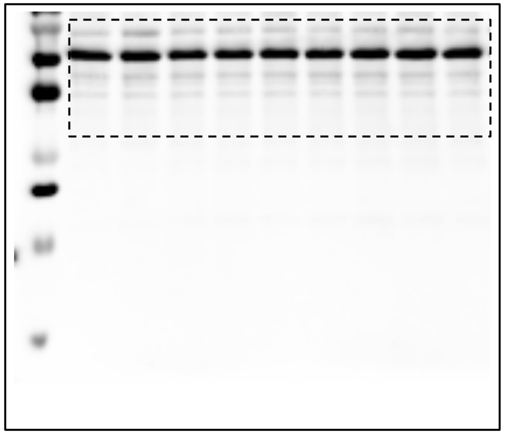

α-Tubulin

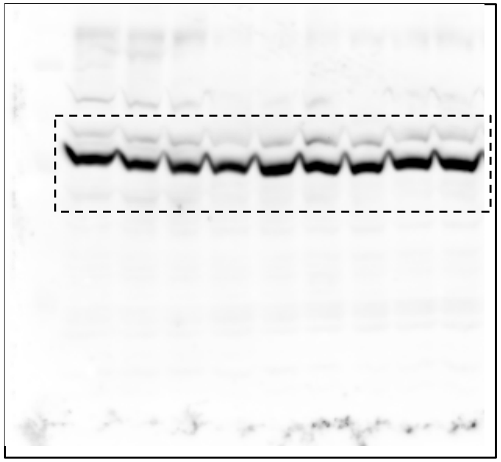

total Akt

Figure S3D Supplementary

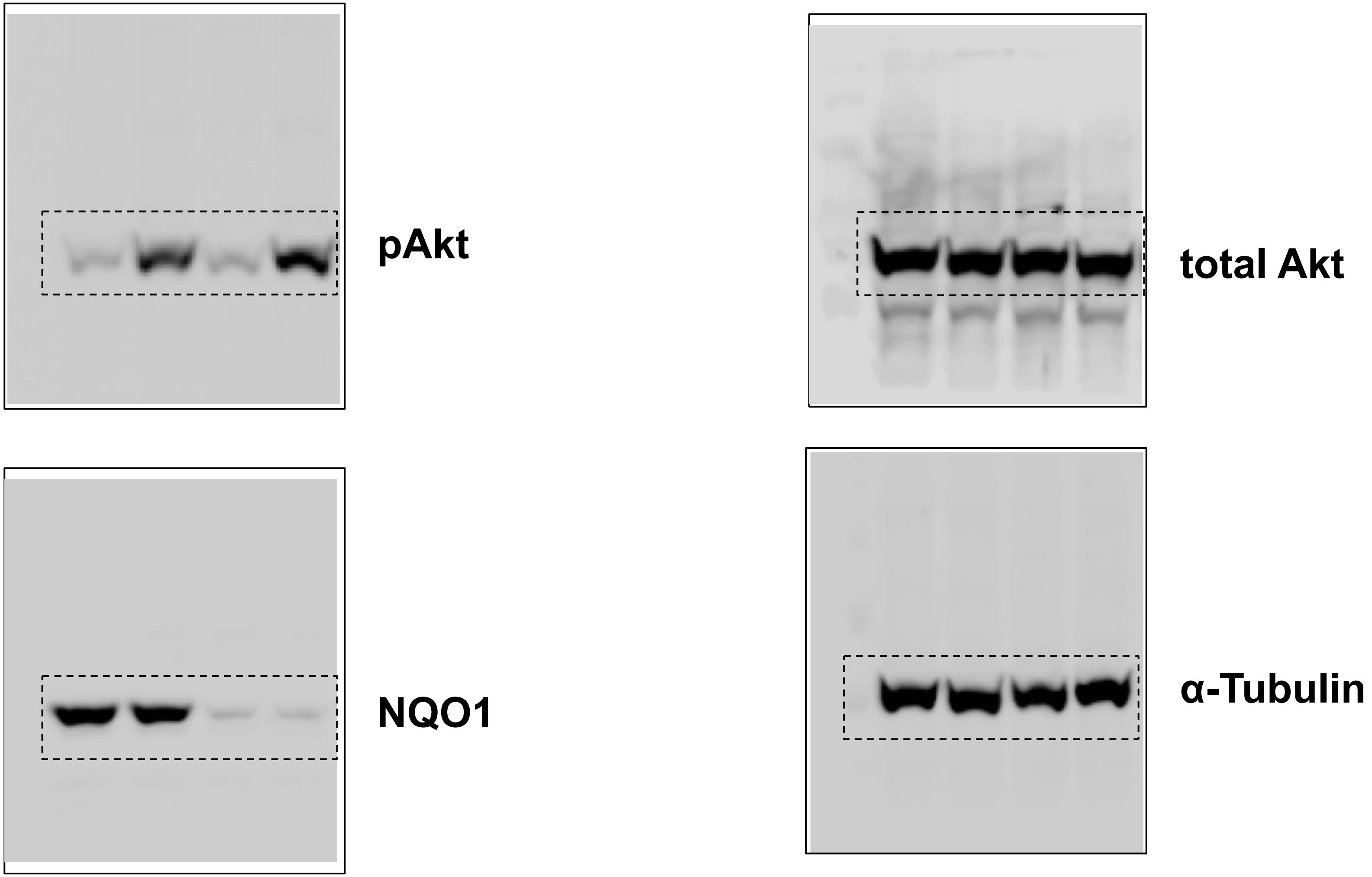

Figure S3E Supplementary

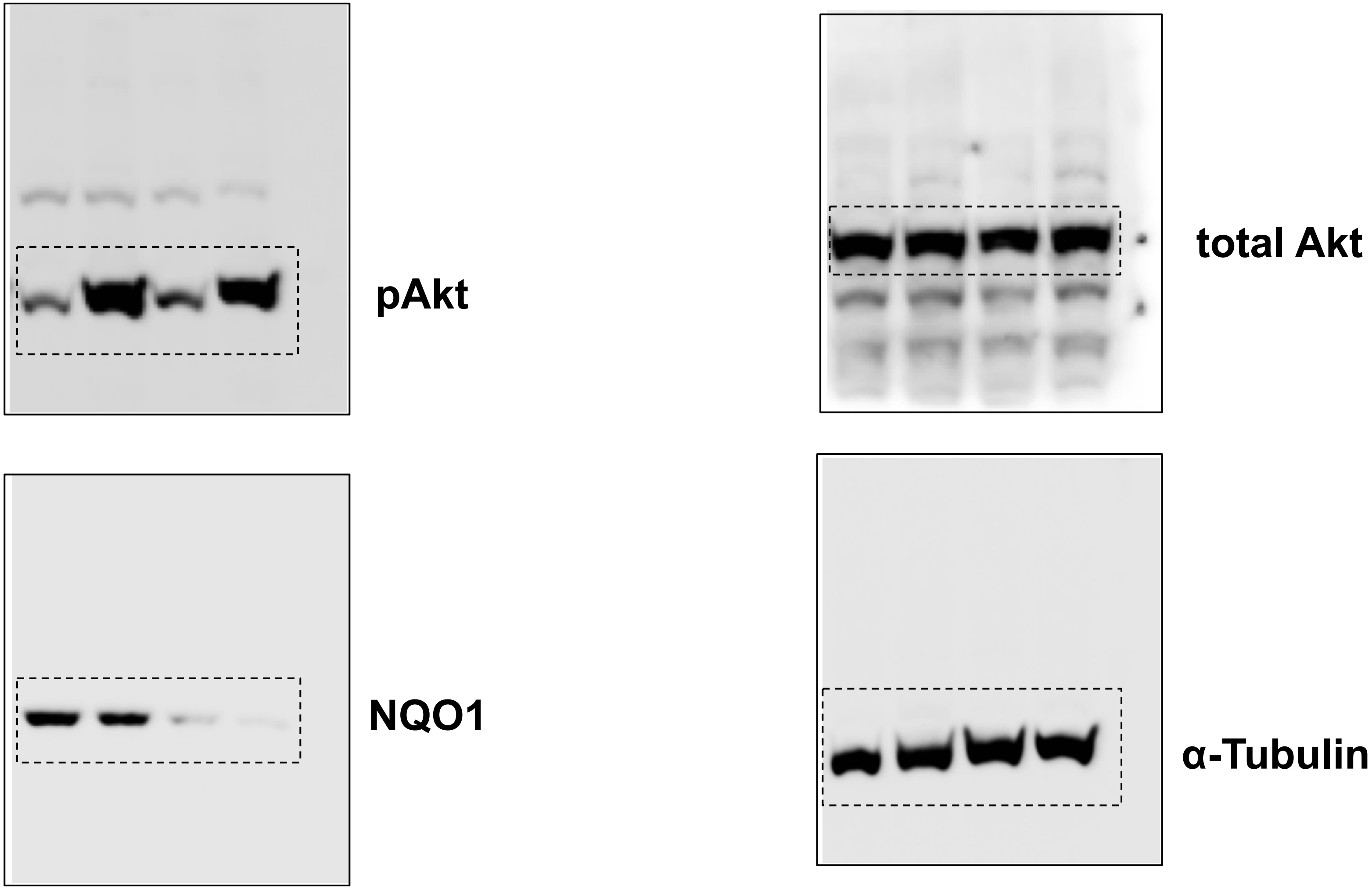

Supplement: Supplementary file 1 — Supplementary Information. [file 41598_2020_76850_MOESM1_ESM.pdf]
